# Systems Biology Analysis of Brucella Infected Peyer's Patch Reveals Rapid Invasion with Modest Transient Perturbations of the Host Transcriptome

**DOI:** 10.1371/journal.pone.0081719

**Published:** 2013-12-09

**Authors:** Carlos A. Rossetti, Kenneth L. Drake, Prasad Siddavatam, Sara D. Lawhon, Jairo E. S. Nunes, Tamara Gull, Sangeeta Khare, Robin E. Everts, Harris A. Lewin, Leslie Garry Adams

**Affiliations:** Department of Veterinary Pathobiology, College of Veterinary Medicine and Biomedical Sciences, Texas A&M University, College Station, Texas, United States of America; Seralogix, Limited Liability Corporation, Austin, Texas, United States of America; Department of Animal Sciences, University of Illinois, Urbana, Illinois, United States of America; University of Osnabrueck, Germany

## Abstract

*Brucella melitensis* causes the most severe and acute symptoms of all *Brucella* species in human beings and infects hosts primarily through the oral route. The epithelium covering domed villi of jejunal-ileal Peyer's patches is an important site of entry for several pathogens, including *Brucella*. Here, we use the calf ligated ileal loop model to study temporal *in vivo Brucella*-infected host molecular and morphological responses. Our results document *Brucella* bacteremia occurring within 30 min after intraluminal inoculation of the ileum without histopathologic traces of lesions. Based on a system biology Dynamic Bayesian Network modeling approach (DBN) of microarray data, a very early transient perturbation of the host enteric transcriptome was associated with the initial host response to *Brucella* contact that is rapidly averted allowing invasion and dissemination. A detailed analysis revealed active expression of Syndecan 2, Integrin alpha L and Integrin beta 2 genes, which may favor initial *Brucella* adhesion. Also, two intestinal barrier-related pathways (Tight Junction and Trefoil Factors Initiated Mucosal Healing) were significantly repressed in the early stage of infection, suggesting subversion of mucosal epithelial barrier function to facilitate *Brucella* transepithelial migration. Simultaneously, the strong activation of the innate immune response pathways would suggest that the host mounts an appropriate protective immune response; however, the expression of the two key genes that encode innate immunity anti-*Brucella* cytokines such as TNF-α and IL12p40 were not significantly changed throughout the study. Furthermore, the defective expression of Toll-Like Receptor Signaling pathways may partially explain the lack of proinflammatory cytokine production and consequently the absence of morphologically detectable inflammation at the site of infection. Cumulatively, our results indicate that the *in vivo* pathogenesis of the early infectious process of *Brucella* is primarily accomplished by compromising the mucosal immune barrier and subverting critical immune response mechanisms.

## Introduction

Brucellosis is a worldwide anthropozoonotic infectious disease caused by small aerobic, non-motile, Gram negative coccobacilli belonging to the genus *Brucella*. The traditional classification of *Brucella* species (*B. melitensis, B. abortus, B. suis, B. canis, B. ovis* and *B. neotomae*) is based on host preference [1]. Recent isolates from human (*B. inopinata*), aquatic mammals (*B. pinnipedialis* and *B. ceti*) and a common vole (*B. microti*) have been recognized as new species [2], [3], [4], bringing the current number to ten species in the genus. In susceptible hosts, *Brucella* spp. produce chronic infections with persistent or recurrent bacteremia, and in middle to late gestation abortion in pregnant animals. With the exception of *B. ovis* and *B. neotomae* that are exclusively pathogenic in their primary hosts (sheep and desert rat wood, respectively), and the newest *Brucella* species whose host specificity has yet to be fully evaluated, brucellae are able to infect other susceptible animals with similar pathogenic effect and clinical disease [5].

The preferred hosts for *B. melitensis* are goats and sheep. However, *B. melitensis* can also infect cattle, among which it can be transmitted under specific epidemiological conditions [6], [7], [8], and it causes the most severe and acute symptoms in human beings [9]. The predominant route for *B. melitensis* penetration after natural exposure is the alimentary tract [1], [10]. Susceptible hosts are primarily infected by contact with aborted fetuses and placental membranes or ingestion of contaminated milk products. Usually *B. melitensis* enter through the oral mucosa and colonize the lymph nodes that drain the eye, nose and mouth [11], however several studies have isolated *Brucella* from different sections of the alimentary tract [12] and feces [13] revealing that brucellae survive under the different environmental conditions of the alimentary canal and invade in multiple sites of the gastrointestinal tract.

The epithelium covering domed villi of jejunal-ileal Peyer's patches is an important site of entry for several pathogens, including *Brucella* [12], [14]. The calf ligated ileal loop model has demonstrated to be a very useful model to study *in vivo* host:agent molecular and morphological initial interaction [15], [16], [17], [18], a field of study that has been somewhat neglected in brucellosis. We hypothesize that in the early phase of infection *B. melitensis* actively modulates host responses to avert pathological lesions and immune-based inflammatory cellular pathways to rapidly establish bacteremia and colonize reticular-endothelial and reproductive systems. Here, we describe the temporal *in vivo* transcriptional profile of the bovine jejunal-ileal Peyer's patches after 0.25, 0.5 1, 2 and 4 h post-*B. melitensis* infection based on a systems biology Dynamic Bayesian Network modeling approach (DBN) of microarray data. Our results document *Brucella* bacteremia occurring within 30 min after intraluminal ileum inoculation without histopathologic traces of lesions and only a transient, very early perturbation of the host enteric transcriptome associated to the initial host:pathogen interactions that was rapidly averted later by the pathogen. These data identify major perturbations of pathways/GO and mechanistic regulatory points modulating critical cellular elements at the onset of the *Brucella* infectious process.

## Materials and Methods

### Bacterial strain, media and culture conditions

Smooth virulent *Brucella melitensis* 16M Biotype 1 (ATCC 23456) (American Type Culture Collection, Manassas, VA), originally isolated from an aborted fetal goat was maintained as frozen glycerol stocks. An aliquot of a saturated culture was inoculated into a 50 ml of cell culture media [F12K medium (ATCC)] supplemented with 10% heat-inactivated fetal bovine serum (HI-FBS) (ATCC)], and incubated at 37°C with 5% CO2 with shaking (200 rpm) until reaching the late-log growth phase (OD600  = 0.4) [19]. The concentration and purity of the inoculum was confirmed by plating a serial dilution on tryptic soy agar (TSA) (BD, Franklin Lakes, NJ) and incubated at 37°C with 5% CO2.

### Experimental animals and ligated ileal loop surgeries

Four unrelated, clinically healthy 3 to 4-week old, brucellosis-free, male Holstein calves weighing 45–55 kg, maintained on antibiotic-free milk replacer twice daily up to 24 h and water *ad libitum* up to 12 h prior to the surgery, were used in these experiments. To minimize the interference that other enteropathogens may have on the host gene expression profile, all calves were tested for fecal excretion of *Salmonella* spp. and *Eimeria* spp. oocysts twice as previously explained [15], [17], and only negative animals were used. All animal experiments were approved by the Texas A&M University Institutional Animal Care and Research Advisory Committee (AUP 2003-178). Surgeries were performed under biosecurity laboratory 3 (ABSL3) conditions in CDC approved isolated buildings located in the Veterinary Medical Park, Texas A&M University (College Station, TX). The surgical procedures were previously described by our laboratory [20]. Briefly, the calves were fasted for 12 hours prior to the surgery. The calves were pre-sedated with propofol (Propoflo; Abbot Laboratories, Chicago, IL) followed by placement of an endotracheal tube and maintenance with isofluorane (Isoflo; Abbott Laboratories) for the duration of the experiment. Throughout the experimental procedure (12 h), the calves were monitored for vital signs (blood pressure, heart rate, hydration status, depth of anesthesia and temperature). The abdominal wall was incised, the distal jejunum and ileum exteriorized, and segments ranging in length from 6 to 8 cm were ligated with umbilical tape leaving 1-cm interloops between them. Seven loops were inoculated intraluminally with 3 ml of a suspension containing 1×109 CFU of *B. melitensis* 16 M/ml (*B. melitensis* inoculated loops) using a 26 gauge 3/8 inch needle, and the seven other loops (media inoculated control) were injected with 3 ml of sterile cell culture media (F12K media supplemented with 10% HI-FBS). The loops were replaced into the abdominal cavity, the incision temporarily closed, and reopened for collecting samples beginning at 15 min and continuing through 12 h. Calves were euthanized with an intravenous bolus of sodium pentobarbital at the completion of the procedures. One infected and one control loop were collected at 7 time points (0.25, 0.5, 1, 2, 4, 8 and 12 h post-inoculation), and the samples were processed for quantification of tissue-associated bacteria, morphology and host gene expression profiling. Samples from the surgery room were transported in triple container to an approved BSL3 laboratory for immediate processing.

### Bacteriology

For quantification of Peyer's patch-associated *B. melitensis*, two-6 mm biopsy punches (0.1 g) were excised from every infected loop, intensely washed three times in PBS to reduce extracellular bacteria, macerated and diluted in 1 ml of distilled water. Similar procedures were followed in control loops to identify any viable *Brucella*, cross contamination during material processing, or *Brucella* dissemination via lymphatic or blood vessels. To determine the number of viable CFU of *B. melitensis* in tissues, lysates were serially diluted and cultured on selective Farrell's selective media [TSA (BD) supplemented with *Brucella* selective supplement (Oxoid Limited, Hampshire, UK) according to manufacture's instructions] [21].

To address the possibility of *B. melitensis* systemic invasion and dissemination, 5 ml of blood were collected by aseptic venipuncture of the jugular vein into 0.75 ml of acid-citrate-dextrose (ACD) at T0 (pre-inoculation), 0.5, 1, 2, 4, 8 and 12 h time, points, and samples from mesenteric lymph nodes and liver were immediately extracted after the calves were euthanized. One ml of blood from every time point was cultured in a non-selective biphasic medium (also known as Castañeda's media) [21]. Briefly, TSA (BD) +1% agar (Difco, Lawrence, KS) cooled to 56°C after autoclaving before adding the *Brucella* selective supplement (Oxoid). The molten medium was well mixed and 20 ml dispensed into 75 cm2 cell culture flask (Corning, Corning, NY). Flasks were laid down to allow the media to solidify along one side. The following day, tryptic soy broth (TSB) (BD) was autoclaved, cooled and *Brucella* selective supplement (Oxoid) added according to manufacture's instructions. Fifteen ml were dispensed aseptically in each flask already containing the solid phase, and the sterility of the media was checked by overnight incubation at 37°C. Blood cultures were incubated for at least 1 month at 37°C and examined for growth of *Brucella* twice a week. To quantify the number of viable CFU of *B. melitensis* in mesenteric LN and liver, 0.1 g of tissue were lysed, serially diluted in distilled water and cultured on selective Farrell's selective media.

### Morphologic analysis

For light microscopy observation, full cross-sections of each loop always including Peyer's patch were fixed in buffered 10% formalin, processed according to the standard procedures for paraffin embedding, sectioned at 5-µm thickness, stained with hematoxylin and eosin, and examined with light microscopy.

### Isolation of total RNA from intestinal loops

Six to ten 6 mm biopsy punches were excised from every loop. The mucosa of the samples was immediately dissected, macerated and homogenized in TRI-Reagent® (Ambion, Austin, TX) (2 biopsy punches/1 ml of reagent) with a hand-held mechanical tissue grinder equipped with a RNase, DNase free plastic disposable pestle. RNA was extracted according to TRI-Reagent manufacturer's instructions. The pellet was re-suspended in DEPC-treated water (Ambion) with 2% DTT and 1% RNase inhibitor (Promega). Contaminant genomic DNA was removed by RNase-free DNase I treatment (Ambion) according to the manufacture's instructions, and samples were stored at −80°C until used. RNA concentration was quantified by NanoDrop® ND-1000 (NanoDrop, Wilmington, DW), and the quality was determined using a Agilent 2100 Bioanalyzer (Agilent, Palo Alto, CA).

### Sample preparation and slide hybridization

Four biological replicates from every time point (T0.25, T0.5, T1, T2 and T4) and every condition (*B. melitensis*- inoculated loops and media-inoculated control loops) were labeled and hybridized as previously described [22]. Briefly, 10 µg of total RNA from each experimental sample (*n* = 40) were reverse transcribed to cDNA using random hexamer primers (Invitrogen), labeled with Cy5 (Amersham Pharmacia Biosciences) and co-hybridized against Cy3 labeled cDNA generated from the bovine reference RNA sample to a custom 13K bovine 70mer oligoarray [23]. Slides were hybridized at 42°C for approximately 40 h in a dark humid hybridization chamber (Corning).

### Microarray data acquisition, normalization and analysis

Immediately after washing, the slides were scanned using a commercial laser scanner (GenePix 4100; Axon Instruments Inc., Foster City, CA). Scans were performed using the autoscan feature with the percentage of saturated pixels set at 0.03%. The genes represented on the arrays were adjusted for background and normalized to internal controls using image analysis software (GenePixPro 6.0; Axon Instruments Inc.). Genes with fluorescent signal values below background were disregarded in all analyses. Arrays were initially normalized against the bovine reference RNA, and the resulting data were analyzed and modeled using an integrated platform termed the BioSignature Discovery System (BioSignatureDS™) (Seralogix, LLC, Austin, TX; www.seralogix.com) explained in detail elsewhere [15], [17], [24], [25], [26]. To achieve a rigorous analysis, genes were ranked and ordered according to their expression magnitudes and gene variance was computed using a Bayesian predicted variance value. The Bayesian variance was determined by using a sliding window algorithm that averages 50 variances directly on the ascending and descending ordered sides of each gene of interest [27]. Significantly changed genes were determined with the Bayesian *z*-test (*p*<0.025). BiosignatureDS tools for statistical Z-score gene thresholding, Bovine pathway and GO activation scoring, Mechanistic gene identification and Genetic network system model were used for the comprehensive analysis performed in this study. Microarray data are deposited in the Gene Expression Omnibus at the National Center for Biotechnology Information (http://www.ncbi.nlm.nih.gov/geo/) Accession # GSE41835.

### Microarray results validation

Five immunity-related genes, which had differential expression by microarray results, were analyzed at every time point by quantitative RT-PCR (qRT-PCR) following the protocol described elsewhere [15], [17]. Briefly, two micrograms of RNA were reverse transcribed using TaqMan® Reverse Transcription reaction (Applied Biosystems, Foster City, CA). For relative quantitation of target cDNA, samples were analyzed in individual tubes in SmartCycler II (Cepheid, Sunnyvale, CA). Primers (Sigma Genosys) of tested genes were designed by Primer Express Software v2.0 (Applied Biosystems) (**Table 1**). For each gene tested, the individual calculated threshold cycles (Ct) were averaged among each condition and normalized to the Ct of the bovine glyceraldehyde phosphate dehydrogenase (GAPDH) from the same cDNA samples before calculating the fold change using the ΔΔCt method [28]. Statistical significance was determined by Student's *t*-test and expression differences considered significant when *P*<0.05. As gene expression by microarray and qRT-PCR were based on *z*-score and fold-change, respectively, array data were considered valid if the fold change of each gene tested by qRT-PCR was expressed in the same direction as determined by microarray analysis.

**Table 1:** Primers for Real Time – PCR Analysis of Genes in *B. melitensis*-Infected Bovine Peyer's Patch.

GENEBANK ACCESSION #	GENE SYMBOL	GENE NAME	FORWARD PRIMERS (5′-3′)	REVERSE PRIMERS (5′-3′)
NM_173895	*BPI*	Bactericidal/permeability-increasing protein	CCTCCGAAACTCACCATGAAG	TGTCCAATCTGAGCTCTCCAATAA
NM_175793	*MAPK1*	Mitochondrial-activated protein kinase 1	GGCTTGGCCCGTGTTG	GGAAGATGGGCCTGTTGGA
NM_174006	*CCL2*	Chemokine (C-C motif) ligand 2	TCCTAAAGAGGCTGTGATTTTCAA	AGGGAAAGCCGGAAGAACAC
NM_173925	*IL8*	Interleukin 8	TGCTTTTTTGTTTTCGGTTTTTG	AACAGGCACTCGGGAATCCT
NM_001033608	*MIF*	Macrophage migration inhibitory factor	CTGCAGCCTGCACAGCAT	TTCATGTCGCAGAAGTTGATGTAG
NM_001034034	*GAPDH*	Glyceraldehyde-3-phosphate dehydrogenase	TTCTGGCAAAGTGGACATCGT	GCCTTGACTGTGCCGTTGA

## Results and Discussion

### *B. melitensis* Colonize and Invade Bovine Host through Peyer's patches Without Inducing Morphologic Lesions in the First 12 h p.i

While it is accepted that the alimentary tract is the main route of invasion for *B. abortus* and *B. melitensis* [1], [10], however the specific portal(s) of entry are not well defined. Although *Brucella* spp. are not considered to be enteric pathogens [29], *B. abortus* was isolated from the small intestine of calves 5 h after being fed with infected milk [12] and from feces of coyotes orally infected [13], which indicates that under natural conditions viable *Brucella* are able to reach the intestinal Peyer's patches.

**Figure 1:**
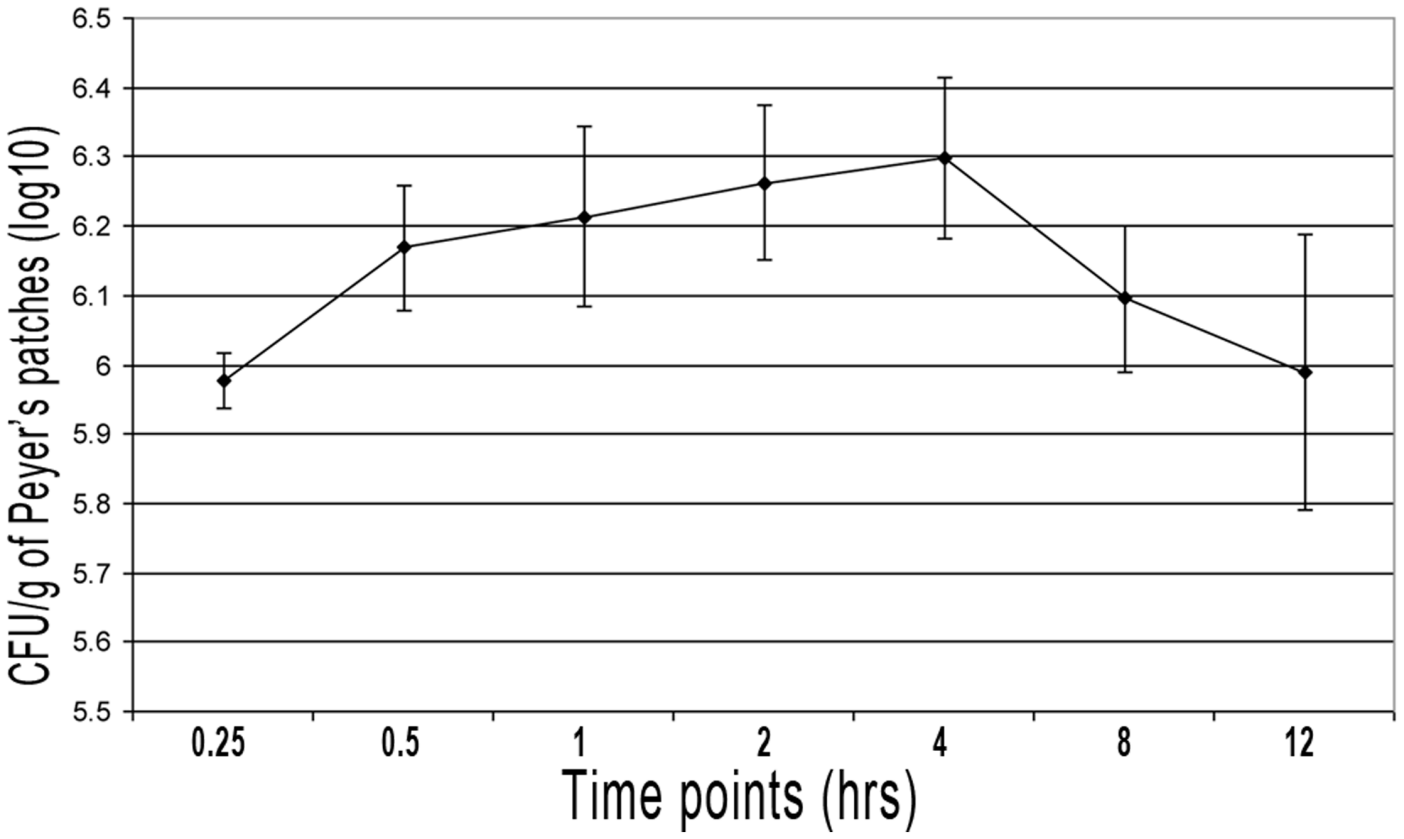
Kinetics of Peyer's Patch Infection with *B. melitensis* 16 M. Jejunal-ileal loops were intraluminally inoculated in 3 ml containing 1×109 CFU of *B. melitensis* 16 M/ml. Tissue (Peyer's patch) samples of 0.1 g of mucosal tissue were extracted at 0.25, 0.5, 1, 2, 4, 8 and 12 h from every infected loop, intensively washed 3 times in PBS, macerated and diluted in 1 ml of distilled water. To evaluate the kinetics of the infection, macerated samples were serially diluted and cultured on Farrell's medium. Numbers of CFU recovered from bovine Peyer's patches are the average of 4 calves. Bars represent standard deviation.

We assessed the kinetics of *B. melitensis* 16 M infection after intraluminal inoculation by quantifying the CFU present in the Peyer's patches at different time points. The intestinal loops were intraluminally inoculated with 3×109 CFU of *B. melitensis* 16 M and 0.1 g of Peyer's patches collected beginning at 15 min and continuing through 12 h. Fifteen minutes post inoculation (p.i.), a few less than 106 CFU/g of tissue were recovered. The number of tissue-associated *B. melitensis* rose rapidly, reaching the peak (2×106 CFU/g of tissue) by 4 h p.i., and decreasing at later time points (**Figure 1**). No differences in histopathological features were detected between control loops and *B. melitensis*-infected loops of the four calves at any time during the 12 h p.i. experiments. The inverse relation between *Brucella* invasion and time post-infection was also observed in *B. abortus* S19-infected bovine ileal loops [30]. The decreasing uptake over the time was attributed to the degeneration of *Brucella* in the intestinal lumen and consequent lack of adhesion due to enteric surface modifications. Another proposed explanation was that *Brucella* saturated the surface receptors on the epithelium, as it was reported in *Salmonella* [31]. A third possible explanation for the decreasing number of tissue-associated *Brucella* over time may be the very rapid translocation from the lumen through the tissue into lymph and ultimately into systemic circulation.

To assess *Brucella* systemic invasion and dissemination, blood from the jugular vein was collected and cultured. Blood samples taken from the first calf were contaminated and not considered in the final analysis of the data. Blood from the second calf was collected as early as 1 h time point while samples from the two other animals were taken from 30 min p.i. through the end of the procedure. All blood samples before the inoculation (T0, control) were *Brucella*-free, but *B. melitensis* were isolated (but could not be quantified) by using Casteñeda's media from all samples from 30 min p.i. through 12 h p.i. *B. melitensis* were also isolated from mesenteric lymph nodes and liver at 12 h p.i. (an average of 3.2×103 and 1×102 *B. melitensis* CFU/g of tissue, respectively) from all inoculated calves, and also from control loops of one animal at 8 and 12 h time points (2×102 and 8×102 CFU of *B. melitensis*/g of Peyer's patch, respectively). These results indicate a very rapid penetration of *B. melitensis* through Peyer's patches followed by systemic dissemination and organ colonization via blood and lymphatic vessels without traces of associated histopathologic lesions.

To elucidate host and *Brucella*-induced host mechanisms responsible for this very rapid invasion and translocation, we studied global gene expression using microarray analysis to host gene expression coupled with a Bayesian inference analysis modeling.

### Host gene expression response is perturbed immediately after infection but rapidly returns to normal state

**Figure 2:**
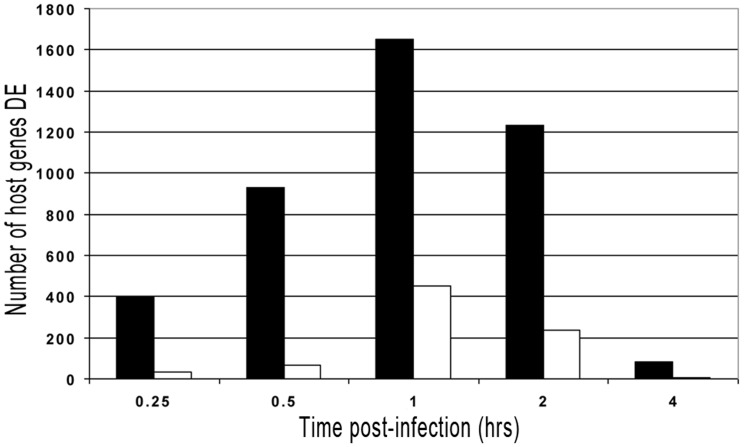
Graphic Representation of Bovine Genes Differentially Expressed throughout the Experiment. (DE  =  differentially expressed). Solid bars represent genes up-regulated; open bars represent genes down-regulated.

**Figure 3:**
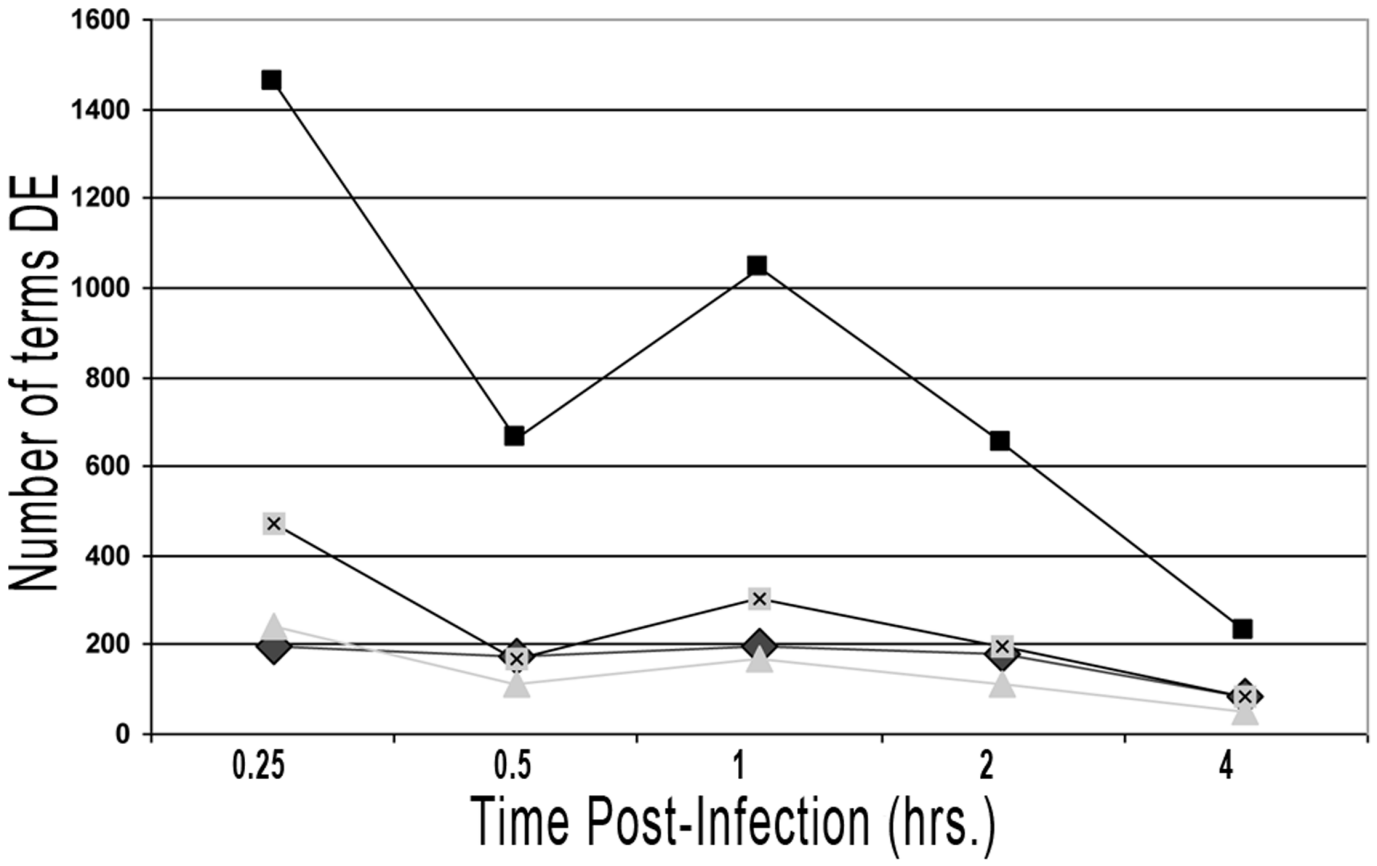
Graphic Representation of Number of Pathways and GO Terms Differentially Expressed at each time point from 15 min to 4 h p.i. Squares  =  GO Biological processes; Squares with X  =  GO Molecular functions; Triangle  =  Cellular components; Diamond  =  Pathways.

Previous work in our lab indicated that four is the lowest number of biological replicates (i.e., calves) to detect significant differences between the treatments used [15], [17], [25]. Considering that *B. melitensis* was isolated from control intestinal loops of one animal at 8 and 12 h p.i., only RNA samples taken from 15 min to 4 h p.i. were considered for further analysis. Bayesian inference identified a progressive host gene, pathway and gene ontology (GO) terms expression modification with the highest activity at one hour p.i. in infected intestinal loop tissues compared with control tissues, which decreased at later time points (**Figure 2** and 3). More details are shown in **File S1, Supplementary Figure 1 and Supplementary Table 1 through 23 (TableS1 – S23 in File S1**). Overall, these results indicate that host molecular response is markedly perturbed at a very early time post-*Brucella* infection, with a strong tendency to rapidly return to a normal state at later time points. These results are in concordance with results from our previous experiments [26] and other studies [36] that had analyzed host gene expression at two different time points after *Brucella* infection.

Focusing our analysis on host gene expression alterations involved in response to *Brucella* spp. infection [22], [26], [32], [33], [34], [35], [36] has enabled us to follow the behavior of pathways for Cell communications, Cell growth and death, Cell motility, Immune system, Infectious disease, Membrane transport, Signal transduction and Signaling molecules and interaction categories. These categories create a subgroup of 56 pathways.

**Figure 4:**
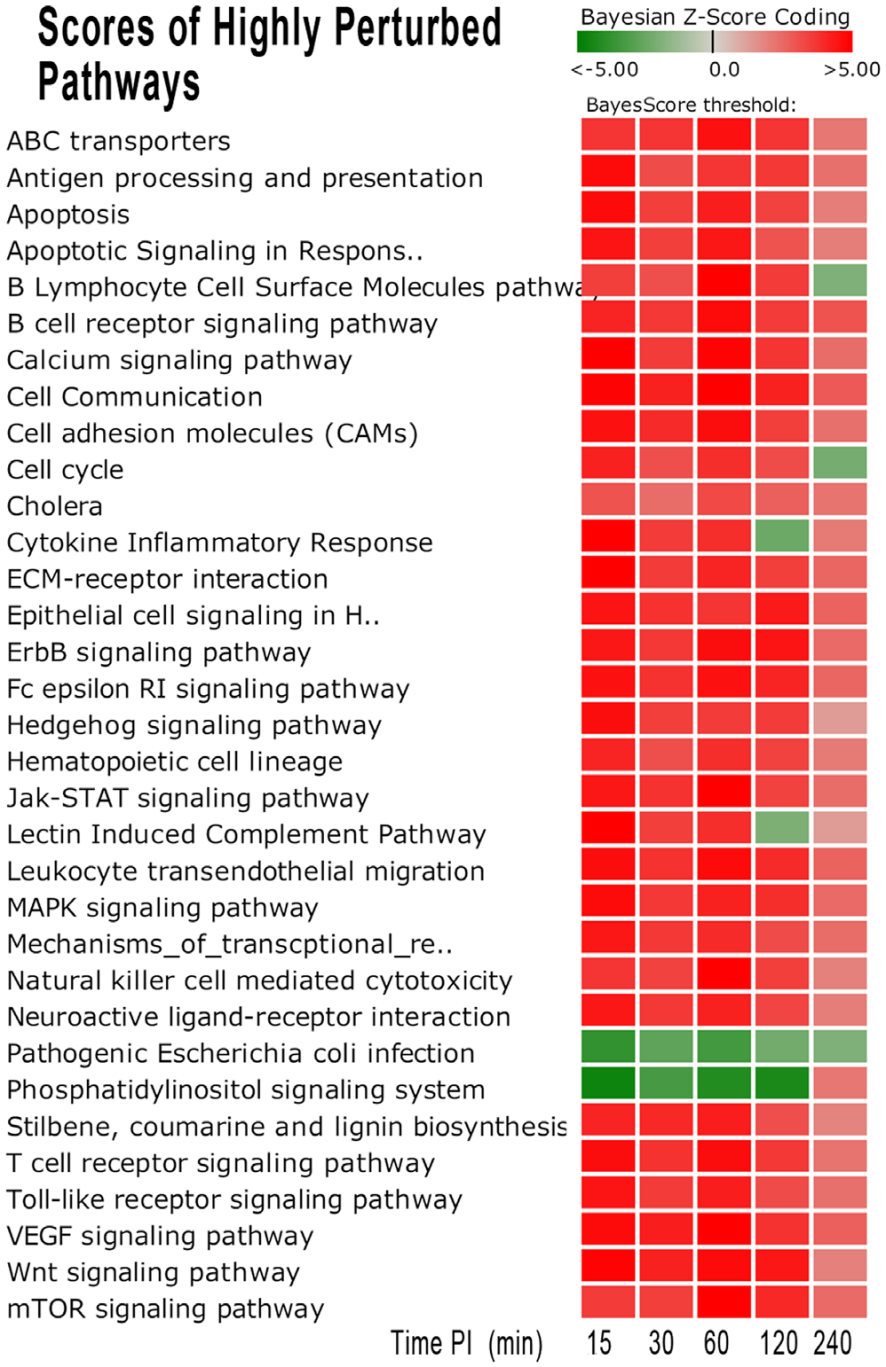
Heatmap of Significantly Perturbed Pathways Scores in Bovine Peyer's Patch Infected with *B. melitensis*. Thirty-seven pathways were identified as significantly perturbed (97.5% confidence) from the early stage of infection (15–60 m.p.i.). The darker red gradients indicate higher Bayesian activation scores (more up-regulated gene expression within the pathway) while the darker green gradients indicate more repressed pathway activity (more down-regulated gene expression).

Among them, there were 37 pathways that were highly perturbed at earlier time points (either activated or repressed) and then had decreased differential expression (DE) at later time points to near or non-statistical differences in comparison with control tissues (**Table 2**, Figure 4). Within these perturbed (either activated or repressed) pathways, there were several pathways that have been extensively studied in host-response manipulation by *Brucella* infection such as Apoptosis [37], [38], Antigen processing and presentation [39], [40], Toll like receptor signaling [14], Leukocyte transendothelial migration [41] and MAPK signaling [26], [42].

**Table 2:** Bayesian *z*-score of 56 Pathways in Eight categories post- *B. melitensis* Infection Compared to Controls.

Name	Description	Category*	0.25 h	0.5 h	1 h	2 h	4 h
hsa04510	**Integrin-mediated cell adhesion**	CC	4.48	3.68	4.3	4.35	2.31
hsa04520	Adherens junction	CC	4.06	−3.13	4.01	3.84	1.84
hsa04530	**Tight junction**	CC	−4.35	−3.68	−4.74	4.27	2.23
hsa04540	**Gap junction**	CC	3.74	2.88	3.05	3.12	2.18
hsa04110	**Cell cycle**	CGD	4.18	3.05	3.86	3.19	−2.21
hsa04210	**Apoptosis**	CGD	4.72	3.44	4.25	3.42	2.02
hsa04810	**Regulation of actin cytoskeleton**	CM	4.51	3.43	4.4	4.16	2.55
hsa04610	Complement and coagulation cascades	IS	4.85	−3.23	4.56	−2.39	2.28
hsa04612	**Antigen processing and presentation**	IS	4.72	3.15	3.69	3.67	2.27
hsa04620	**Toll-like receptor signaling pathway**	IS	4.48	3.55	4.28	3.13	2.29
hsa04640	**Hematopoietic cell lineage**	IS	4.11	3.1	3.87	3.38	2.07
hsa04650	**Natural killer cell mediated cytotoxicity**	IS	3.71	3.36	5.13	3.5	1.9
hsa04660	**T cell receptor signaling pathway**	IS	4.66	3.82	4.61	3.6	2.2
hsa04662	**B cell receptor signaling pathway**	IS	4.13	3.63	4.72	3.55	2.98
hsa04664	**Fc epsilon RI signaling pathway**	IS	4.56	3.75	4.56	4.08	2.53
hsa04670	**Leukocyte transendothelial migration**	IS	4.65	3.77	4.73	3.91	2.58
hsa99103	Apoptotic DNA fragmentation and homeostasis pathway	IS	−1.72	−2.16	−2.17	−1.83	−2.18
hsa99104	**Apoptotic Signaling Response to DNA Damage pathway**	IS	4.52	3.47	4.41	3.01	1.98
hsa99105	BRC signaling pathway	IS	3.8	−2.48	3.72	2.19	−1.47
hsa99106	B Cell Receptor Complex pathway	IS	2.39	4.05	4.56	0.95	2.9
hsa94660	**T cell receptor signaling pathway Antigen Processing**	ID	4.71	4.17	4.94	3.73	2.15
hsa00626	Nitrobenzene degradation	ID	4.11	2.21	4.86	2.7	1.69
hsa00940	**Stilbene, coumarine and lignin biosynthesis**	ID	4.13	4	4.28	3.07	1.8
hsa01430	**Cell Communication**	ID	4.92	4.19	5.77	4.16	2.82
hsa05110	**Cholera**	ID	2.98	2.42	3.22	2.67	2.26
hsa05120	**Epithelial cell signaling in Helicobacter pylori infection**	ID	4.47	3.79	3.69	4.35	2.58
hsa05130	**Pathogenic Escherichia coli infection**	ID	−3.77	−2.77	−3.12	−2.7	−2.03
hsa99020	**Lectin Induced Complement Pathway**	ID	5.39	3.48	3.87	−2.06	1.27
hsa99100	Activation of Csk through the T-Cell Receptor pathway	ID	3.29	1.51	2.45	1.75	0.91
hsa99101	Activation of cAMP-dependent protein kinase PKA pathway	ID	2.47	−1.03	1.22	2.29	1.79
hsa99102	Anthrax Toxin Mechanism of Action pathway	ID	2.85	1.39	3.15	3.87	0.99
hsa99107	**B Lymphocyte Cell Surface Molecules pathway**	ID	3.51	3.06	5.2	3.57	−2.01
hsa99108	Blockade Neurotransmitter Release by Botulinum Toxin path	ID	−0.96	0	0	0.63	0
hsa99109	CCR3 signaling in Eosinophils pathway	ID	4.78	−2.89	5.06	−4.63	3.29
hsa99110	CD40L Signaling Pathway	ID	5.71	4.32	3.64	−4.24	3.28
hsa99111	Calcium Signaling by HBx of Hepatitis B virus pathway	ID	−1.23	−0.97	−2.21	−0.1	0
hsa99112	Cytokine Inflammatory Response	ID	5.43	3.58	3.84	−2.49	2.08
hsa99113	Trefoil Factors Initiate Mucosal Healing	ID	−4.46	−3.07	4.37	3.56	−2.57
hsa99114	**Transcptional_repression_by_DNA_methylation**	ID	4.39	3.61	3.91	3.15	2.35
hsa02010	**ABC transporters**	MT	3.74	3.72	4.55	3.7	2.18
hsa04010	**MAPK signaling pathway**	ST	4.75	3.63	4.17	3.89	2.43
hsa04012	**ErbB signaling pathway**	ST	4.39	3.66	4.61	4.53	2.47
hsa04020	**Calcium signaling pathway**	ST	4.97	3.59	4.86	3.68	2.37
hsa04070	**Phosphatidylinositol signaling system**	ST	−4.62	−3.35	−4.21	−4.3	2.18
hsa04150	**mTOR signaling pathway**	ST	3.55	3.46	5.24	4.01	2.45
hsa04310	**Wnt signaling pathway**	ST	4.85	4.15	4.73	4.41	1.93
hsa04330	Notch signaling pathway	ST	−4.43	3.61	3.56	4.11	2.6
hsa04340	**Hedgehog signaling pathway**	ST	4.66	3.49	3.55	3.52	1.27
hsa04350	TGF-beta signaling pathway	ST	4.47	−2.88	−3.65	4.02	1.96
hsa04370	**VEGF signaling pathway**	ST	4.75	4.24	5.11	3.82	2.68
hsa04630	**Jak-STAT signaling pathway**	ST	4.43	3.76	4.93	3.37	2.39
hsa04060	Cytokine-cytokine receptor interaction	SM	4.34	3.38	4.12	−3.06	2.04
hsa04080	**Neuroactive ligand-receptor interaction**	SM	4.44	3.62	4.17	3.31	2.02
hsa04512	**ECM-receptor interaction**	SM	5.03	3.55	4.07	3.47	2.52
hsa04514	**Cell adhesion molecules (CAMs)**	SM	4.55	3.95	4.64	3.48	2.31

* Categories - CC =  cell communication; CGD =  cell growth and death; CM =  cell motility; IS =  immune system; ID =  infectious disease; MT =  membrane transport; ST =  signal transduction; SM =  signaling molecule measured time points 15, 30, 60, 120, and 240 minutes p.i. The Bayeian *z*-scores represent the degree of perturbation of the group of pathway genes versus the controls. Positive *z*-scores represent activation of the pathway (pathway score is dominated by more up-regulated genes), while the negative *z*-score represents pathway repression (pathway score is dominated by down-regulated genes). **Bolded** pathways represent pathways highly perturbed at earlier time points (either activated or repressed) with decreased differential expression at later time points to near or non-statistical differences in comparison with control tissues.

We will now focus the manuscript on the most relevant findings detected by our analysis related to the host response during the early onset of *Brucella* infection, i.e., bacterial adherence, invasion, colonization and dissemination within the host [43], [44].

### Brucella adhere and invade by compromising the mucosal barrier

The bacterial adhesion to cell membrane is the essential first step in colonization, followed by invasion. According to previous publications, *Brucella* attach to cultured epithelial cell lines via receptor molecules containing sialic acid or sulphated residues and also bind extracellular matrix proteins (ECM), which probably contribute to the spread of the pathogen and tissue colonization [45]. A very recent publication indicates that *Brucella* attach to the cellular prion protein on the M cells surface before internalizing [46]. Our analysis identified two adherence-related pathways significantly activated throughout the experiment in infected samples compared to control ones: the Cell Molecules Adhesion (CAM) and the ECM receptor interaction pathways. Deeper analysis of both pathways revealed several genes differentially expressed (Bayesian *z*-score >|2.24|) at different times post-infection (**Tables 3 and 4**). Some of these genes showed an erratic expression, probably due to the several different cell types analyzed simultaneously; however other genes were consistently up or down-regulated through the study and may be strong candidates involved in *Brucella* adherence. Among them, there are specifically four gene products (*SDC2* –syndecan 2, *ITGAL* –integrin alpha L, *ITGB2* –integrin beta 2, and *IBSP* – integrin binding sialoprotein-) that commonly are expressed on cell surfaces and their products are involved in bacterial pathogenesis. Syndecans comprise a major family of cell surface heparan sulfate proteoglycans (HSPGs) and their role in microbial infections is currently an active area of research. *Neisseria gonorrhoeae*, *Pseudomona aeuruginosa* and several enterobacteria attach and invade hosts by interacting to sydecans molecules [47], [48], [49]. Particularly, syndecan-2 (*SDC2*) expressed on the surface of dendritic cells has been shown to bind to HIV and facilitate viral transmission to CD4-positive T cells [50]. Until now, nothing was known about the role of SDC in *Brucella* pathogenesis, but this finding may illustrate a fruitful avenue of future investigation. Integrins are a group of mammalian cell surface molecules which can function as receptors for bacterial ligands, promoting bacterial adherence and internalization into mammalian cells [51]. ITGAL combines with ITGB2 to form the integrin LFA-1 (lymphocyte function-associated antigen-1), which is expressed on all leukocytes and resting Langerhan's dendritic cells as well [52]. We had demonstrated previously that blocked α-chain (ITGAL) of the integrin LFA-1, decrease *Brucella abortus* binding to mononuclear phagocytes [53]. Our studies surprisingly discovered IBSP gene as being mechanistic in nature. IBSP is a major structural protein of the bone matrix, and the only known extraskeletal site of its synthesis is the trophoblast, a major target cell for the pathogenesis of *Brucella* induced abortion. Little is known about the potential roles of the IBSP gene product in the microbial pathogenesis of brucellosis particularly in Peyer's patch, however given the uniqueness of this finding IBSP deserves special emphasis.

**Table 3:** Significantly Altered Genes (Bayesian *z*-score |2.24|) in the Cell Molecule Adhesion (CAM) pathway.

Symbol	Description	0.25 h	0.5 h	1 h	2 h	4 h
Cd28	CD28 antigen	2.12	0.69	−3.13	0	−0.22
CD34	CD34 molecule	0	−1.61	−2.02	−2.35	0
CD58	CD58 molecule	−3.47	−2.7	−2.81	−3.13	0
CD99	CD99 molecule	−0.99	0.86	0.82	2.5	0
CDH1	cadherin 1, type 1, E-cadherin (epithelial)	−1.88	−2.82	0	−1.83	0.52
CDH15	cadherin 15, M-cadherin (myotubule)	2.42	0.66	−0.59	2.99	1.63
CDH3	cadherin 3, type 1, P-cadherin (placental)	2.85	1.75	0	0	−0.99
CDH5	cadherin 5, type 2 (vascular endothelium)	−3.36	−2.08	−2.54	−2.65	−1.97
CLDN1	claudin 1	−2.49	−1.73	−2.39	−1.76	−1.03
Cldn16	claudin 16	2.42	1.46	1.05	0.28	2.09
CLDN3	claudin 3	2.71	0	0	0.42	2.27
CLDN4	claudin 4	1.33	2.18	2.43	0.95	1.29
CLDN5	claudin 5 (transmembrane protein deleted in velocardiofacial)	1.79	2.14	2.95	0	1.94
CLDN7	claudin 7	1.16	−0.65	−3.48	−2.05	0
CNTN1	contactin 1	−2.86	−1.18	−1.43	−0.68	−0.21
Cntnap2	contactin associated protein-like 2	0.51	2.59	3.41	2.75	1.26
VCAN	versican	2.7	3.98	5.16	4.14	2.58
ESAM	endothelial cell adhesion molecule	2.94	4.05	1.81	1.85	2.87
VCAM1	vascular cell adhesion molecule 1	−2.33	−2.02	−3.27	−3.89	−1.26
Pecam1	platelet/endothelial cell adhesion molecule 1	2.87	0.8	−1.18	−1.76	−0.64
NCAM1	neural cell adhesion molecule 1	−3.03	0	0	1.22	−0.1
F11R	F11 receptor	−0.74	−0.92	−0.3	−1.37	2.42
GLG1	golgi apparatus protein 1	0.51	1.04	2.22	0.54	2.72
HLA-A	major histocompatibility complex, class I, A	−3.44	−2.13	−2.14	−2.92	0
HLA-DMA	major histocompatibility complex, class II, DM alpha	1.35	3.65	2.3	0.75	0
HLA-DOA	major histocompatibility complex, class II, DO alpha	2.37	0	1.95	0	0.61
HLA-DOB	major histocompatibility complex, class II, DO beta	0.97	0.98	2.45	0.08	1.25
HLA-DRA	major histocompatibility complex, class II, DR alpha	−3.07	−1.86	−1.32	−3.09	−1.57
ICAM1	intercellular adhesion molecule 1	0.25	0	2.25	0	0
ICAM2	intercellular adhesion molecule 2	3.06	4.5	6.19	0.44	2.82
ITGA4	integrin, alpha 4 (antigen CD49D, alpha 4 of VLA-4 receptor)	2.04	2.18	3.61	−0.63	0
ITGA8	integrin, alpha 8	0.47	1.12	3.18	0.22	0.9
ITGA9	integrin, alpha 9	−4.84	−3.42	−2.55	−2.27	−2.02
ITGAL	integrin, alpha L (antigen CD11A (p180), LFA 1; alpha peptide	1.74	2.73	4.28	2.81	0.81
ITGAV	integrin, alpha V (vitronectin receptor, alpha peptide, CD51)	−2.7	−2.42	−0.24	−1.82	0
ITGB2	integrin, beta 2 (complement component 3 receptor 3 and 4 unit)	3.13	2.8	4.15	4.04	2.34
NRXN3	neurexin 3	−2.85	0	−1.88	1.53	−2.39
PTPRM	protein tyrosine phosphatase, receptor type, M	4.37	−3.96	−3.08	−1.19	−0.24
PVRL1	poliovirus receptor-related 1 (herpesvirus entry mediator C)	1.12	1.24	1.05	2.17	2.59
PVRL2	poliovirus receptor-related 2 (herpesvirus entry mediator B)	0.84	1.87	5.18	1.33	1.19
SDC2	syndecan 2	4.05	3.81	3.75	3.27	2.16

The table lists significantly perturbed (activated or repressed) genes at any time among the 5 different time points (0.25 h through 4 h p.i.).

**Table 4:** Significantly Altered Genes (Bayesian *z*-score |2.24|) in the Extra-Cellular Membrane (ECM) pathway.

Symbol	Description	t = 15	t = 30	t = 60	t = 120	t = 240
Agrn	agrin	−0.89	−2.48	1.26	2.28	1.83
Cd47	CD47 antigen (Rh-related antigen, integrin-associated signal transducer)	−3.3	−3.8	−4.64	−1.05	0
Chad	chondroadherin	−2.65	−0.08	−2.44	−1.6	−2.62
Col5a1	collagen, type V, alpha 1	4.03	3.59	4.81	3.31	2.5
Col5a2	collagen, type V, alpha 2	−2.89	−1.59	−1.73	−2.45	−1.94
COL6A2	collagen, type VI, alpha 2	0.13	2.88	2.43	1.84	0.65
COL6A3	collagen, type VI, alpha 3	3.13	0	0	−0.34	1.59
OI4	collagen, type I, alpha 2	0	0	0	−2.95	0
SEDC	collagen, type II, alpha 1	2.35	0	−2.18	−1.74	3.21
DAG1	dystroglycan 1 (dystrophin-associated glycoprotein 1)	−2.29	−0.68	−0.5	0	−0.03
Fndc1	fibronectin type III domain containing 1	4.22	−2.3	−2.69	−0.87	−2.14
Ibsp	integrin binding sialoprotein	−2.82	−2.03	−3.84	−2.43	3.32
ITGA10	integrin, alpha 10	2.86	0.93	4.74	2.05	1.85
ITGA2	integrin, alpha 2 (CD49B, alpha 2 subunit of VLA-2 receptor)	3.18	0.61	1.92	2.26	2.27
ITGA2B	integrin, alpha 2b (platelet glycoprotein IIb of IIb/IIIa complex, antigen CD41)	0.99	2.01	4.05	2.51	2.81
ITGA4	integrin, alpha 4 (antigen CD49D, alpha 4 subunit of VLA-4 receptor)	1.73	1.66	2.88	−0.68	0
ITGA8	integrin, alpha 8	0.36	1.15	3.1	0	0.86
ITGA9	integrin, alpha 9	−3.58	−2.82	−2.38	−1.94	−0.62
ITGB4	integrin, beta 4	1.31	0	2.78	2.22	1.1
LAMA1	laminin, alpha 1	0.38	1.27	3.41	1.28	1.09
Lama5	laminin, alpha 5	−1.32	2.6	0.78	2.42	2.43
LAMB2	laminin, gamma 1 (formerly LAMB2)	0.88	2.67	2.89	2.24	2.48
LOC131873	no description	2.26	1.12	0.12	0.49	0.34
Npnt	nephronectin	3.04	0.04	−0.36	−2.2	0.83
SDC2	syndecan 2	3.71	3.82	3.01	2.91	1.95
THBS1	thrombospondin 1	−3.07	−0.85	0	1.07	1.88
THBS2	thrombospondin 2	2.82	−0.14	−2.71	−2.49	−0.07
Tnn	trophinin	2.89	1.64	0.74	0	0.64
VTN	vitronectin	0	2.25	0	0	−1.33

The table lists significantly perturbed (activated or repressed) genes at any time among the 5 different time points (0.25 h through 4 h p.i.).

**Figure 5:**
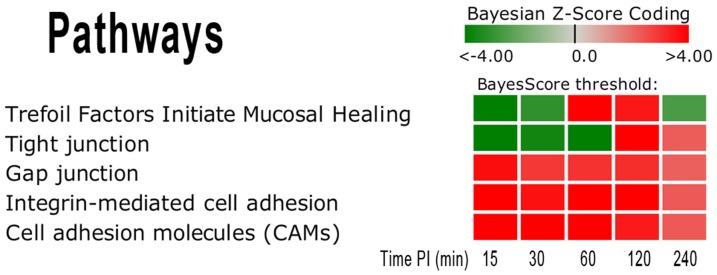
Pathway Score Heat Map for Cell Communication Related Pathways. This heat map shows the repressed state of activity for the Tight Junction (TJ) and Trefoil Factors (TF) pathway in comparison to the Gap Junction, Integrin-mediated Cell Adhesion and Cell Adhesion Molecules pathways. Note that the TJ and TF pathways have a complex activation pattern. The TJ is tri-phasic in that it is highly repressed in the early stage, becomes moderately activated at 60 and 120 min p.i, and then becomes repressed at 240 min p.i. The TF pathway is bi-phasic in that it is highly repressed in the early stage but becomes moderately activated at 120 and 240 min p.i. The darker red gradients indicate higher Bayesian activation scores (more up-regulated gene expression within the pathway) while the darker green gradients indicate more repressed pathway activity (more down-regulated gene expression).

A few minutes after adhesion, *Brucella* are quickly internalized in *in vitro* studies. In non-professional phagocytic cells, *Brucella* are able to auto-induce internalization by activating small GTPases of the Rho subfamily (i.e. Rho, Rac, Cdc42) and modulate rearrangements of the host cell actin cytoskeleton and microtubules [26], [54]. In phagocytic cells, entry and survival of *Brucella* requires functional lipid rafts on cell membrane [55]. *In vivo* studies using the ileal loop model demonstrated that transepithelial migration of *Brucella* occur mainly through endocytosis by the follicle associated epithelium (FAE) of Peyer's patches and uptake by dendritic cells-penetrating the FAE [14], [30], [46]. In addition to enterocytes, a critical component of the intestinal barrier is the intercellular junction complexes between adjacent enterocytes that form a semi-permeable diffusion barrier. Several pathogens have developed strategies to interact and manipulate junctional complexes, in order to disrupt and cross the epithelial barriers [56]. Our pathway analysis revealed two intestinal barrier-related pathways (Tight Junction (TJ) and Trefoil Factors Initiated Mucosal Healing (TFIMH)) significantly repressed in the early stage of infection (15–60 minutes p.i.) (Table 2). The repressed expression of these two pathways associated with *Brucella* host invasion suggest subversion of mucosal epithelial barrier function as previously shown that activation or repression of gene expressions of junction pathways may lead to strengthening or weakening of the primary intestinal barrier, respectively [57], [58]. These results are in concordance with our previous publication where we reported that *Brucella*-infected epithelioid like cells down-regulate GO biological processes related with cell-cell adhesion, such as positive regulation of cell adhesion (GO:0045785), regulation of cell-cell adhesion (GO:0022407), cell-cell junction organization (GO:0045216), regulation of cell adhesion (GO:0030155) and cell junction organization (GO:0034330) among others [26]. Of note is the fact that other cell communication/junction pathways that include Gap Junction (GJ), Adherents Junction (AJ) and Integrin-mediated Cell Adhesion (IMCA) were not scored as repressed as shown in the heat map of **Figure 5**.

#### Tight junction pathway analysis

**Figure 6:**
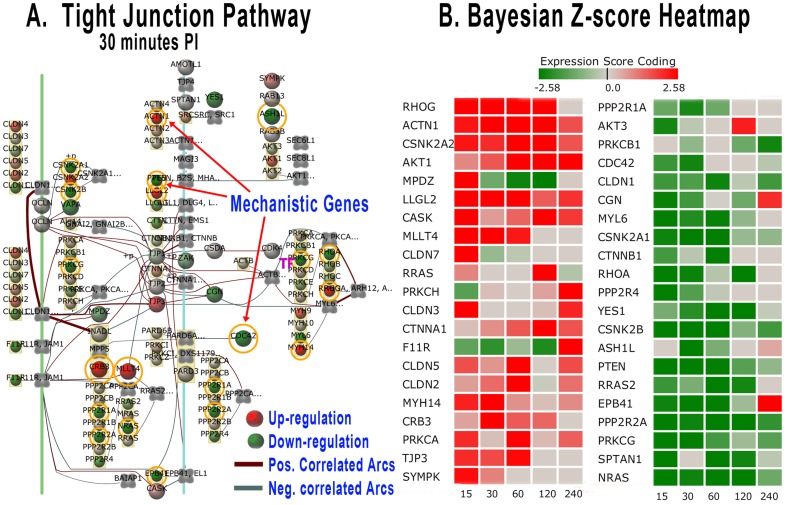
Tight Junction Pathway Bayesian Network Model and Gene Score Heat Map. (A) Tight Junction pathway Bayesian network representation at 30 min post-inoculation. Gene nodes with orange circles on the network are those defined as mechanistic genes that surpass a threshold Bayesian z-score| >2.24|. The network shows gene nodes with gradient colors representing the level of expression (deeper red for higher up-regulated genes and deeper green for down-regulated). (B) The Bayesian score heat map for the gene expression of *Brucella* infected host Peyer's patch versus non-infected controls. The heat map is colorized and corresponds to the gene node expression levels. Grey color represents little to no expression difference between *Brucella*-infected and control loops. The heat map columns are by time post-infection in minutes.

The tight junctions are the most apical intercellular complex and are responsible for controlling the permeability of the paracellular conduits between epithelia cells. The molecular interactions between genes of this pathway were visualized by the BioSignatureDS software as shown in **Figure 6(A)**, while **Figure 6(B)** provides a heat map of genes on this pathway found significantly expressed (Bayesian z-score |>2.24|). The repressed state of the TJ pathway in the first hour p.i. is evident by the numerous down-regulated genes in both the Bayesian network model and the gene score heat map. Some of the more dominant down-regulated genes in the early stage of infection are *NRAS*, *SPTAN1*, *PRKCG*, *PPP2R2A*, *EPB41*, *PTEN*, *CSNK2B*, *YES1*, *RHOA*, *CSNK2A1*, *MYL5*, *CGN*, *CLDN1*, *CDC42*, and *AKT3*. The biological roles of these subverted genes are described in **Supplemental Table 24 (Table S24 in File S1**). Interestingly, *NRAS*, *RHOA*, *CDC42*, and *AKT3* were previously identified as mechanistic genes having intersecting points across several pathways (**Table S22 in File S1**). Of special note are the genes *SPTAN1*, *EPB41*, *RHOA*, *MYL5*, *CGN*, and *CLDN1* that are involved in maintaining the integrity of the epithelial layer and permeability. Additional insight into the regulatory relationships within the Tight Junction pathway was found by interrogating the Bayesian network model of Figure 6(A). The model suggests that *RHOA* (Ras homolog family member A) has significant correlated influence over *MYL6* (myosin, light chain 6, alkali, smooth muscle and non-muscle), which is involved in actin binding. Additionally, it was found that *CGN* (Cingulin) has regulatory influence on the genes *MYL6*, *ACTB* (actin, beta), *MYH9*, *MYH10*, and *MYH14* (myosin, heavy chain 9, 10 and 14), all of which are involved in cell motility, cell shape, integrity and cell attachments.

#### Trefoil factors initiated mucosal healing (TFIMH) pathway analysis

Epithelial continuity can also depend on a family of small, yet abundant, secreted proteins, namely the trefoil factors. The trefoil factors maintain the integrity of the gastrointestinal tract, despite the continual presence of microbial flora and injurious agents [59]. The immune related TFIMH pathway was suppressed in the early stage post infection (**Figure 5**). Unfortunately, not all the trefoil factors gene probes were included on the bovine microarray employed during this study. However, the TFIMH pathway suppression (as determined by other observed gene expressions) implies impaired trefoil factor gene expression, and consequently, a possible invasion mechanism of *Brucella* by subverting mucosal healing. Genes that dominate the suppressed pathway activity are *PTK2*, *GHR*, *RHOA*, *CTNNB1, MAPK3, and SHC1*. The biological roles of these genes are described in **Supplemental Table 25 (Table S25 in File S1)** except for RHOA, which was described in **Table S24 in File S1**. The gene *PTK2* (Protein tyrosine kinase 2) is strongly down regulated across all time points and was identified previously as a mechanistic gene intersecting several pathways (**Table S22 in File S1**). *PTK2* encodes a protein-tyrosine kinase that plays an essential role in regulating cell migration, adhesion, spreading, reorganization of the actin cytoskeleton, formation and disassembly of focal adhesions and cell protrusions, cell cycle progression, cell proliferation and apoptosis. It is found concentrated in the focal adhesions that form between cells growing in the presence of extracellular matrix constituents. Even though *Brucella* are not normally regarded as an enteric pathogens and the paracellular route of entry has not been previously described in its pathogenesis, our analysis indicates that alteration of genes involved in maintenance of the intestinal integrity may constitute an important and novel target associated with the successful invasion by *Brucella* through the host's mucosal barrier.

#### *Brucella* establish and disseminate in the host by subverting the immune response

Upon crossing the first level of defense at the mucosal epithelium, *Brucella* are transported to lymph nodes by phagocytic cells and phagosomes act as intracellular niche for *Brucella* [5]. Based on this premise, it's expected that pathways involved in phagocytosis are activated during *Brucella* infection. Our study confirms that the Lectin pathway, which is initiated by the binding of mannose-binding lectin to carbohydrates found on bacterial cell surfaces and leads to phagocyte recruitment, was significantly activated during the early stage of *Brucella* infection (with a Bayesian *z*-score of >2.24 at 15 to 60 min p.i. [**Table 2**]). This result is in agreement with Fernandez-Prada *et al*. who demonstrated that Lectin pathway was involved in complement deposition and complement-mediated killing of *Brucella* [60]. The Lectin pathway (or mannose-binding pathway) is part of the Complement and Coagulation Cascades (CCC), a nonspecific defense mechanism involved in triggering the innate immune response against pathogens. Of the seven genes in the lectin induced complement pathway, *MASP2*, *C9* and *C5* are up-regulated and *C6* is down-regulated at 15 min p.i.; and *C5* and *C9* are up-regulated at 30 min p.i. The biological roles of these four genes are shown in **Supplemental Table 26 (Table S26** in File S1 **)**. Coincidently, Lectin and CCC pathways were found to be up-regulated in *Brucella*- infected HeLa cells at 4 h post-infection, and *MASP2* gene was indicated as a key mechanistic gene in both pathways [26]. Though the Lectin pathway is significantly activated in the early stages, however, the combined CCC pathway is observed to have temporal cyclic response (activation and repression) during the first 4 h p.i. Activation of the Lectin pathway may lead to phagocytosis, which plays an important role in establishment and dissemination of *Brucella* in the host. The lack of a phagocytic pathway in KEGG, lead us to identify the significant genes for “phagocytosis” using GO terms. Of the 34 genes representing phagocytosis, 13 were significantly perturbed in the first 4 h post-*Brucella* infection. *CD47*, *CDC42SE2*, and *DOCK1* were down-regulated across all time points while *SIRPA* was strongly down-regulated in the early stage of infection. *GATA2* and *AHSG* were up-regulated at 15 min p.i. and *ELMO1*, *ELMO2*, *FCER1G*, *AZU1*, *SCARB1*, *SFTPD* and *CORO1A* were up-regulated between 15–60 min p.i. Their biological roles for phagocytosis GO Group are summarized in **Supplemental Table 27 (Table S27** in File S1 **)**. *CD47* plays an important role in both cell adhesion and in the modulation of integrins, and is a receptor for *SIRPA*, binding to which prevents maturation of immature dendritic cells and inhibits phagocytosis and cytokine production. As previously stated, *SIRPA* was repressed during early *Brucella*-host interaction, but is later activated, suggesting a novel mechanism of *Brucella* manipulation of dendritic cells maturation.

From a different perspective of phagocytosis, Starr *et al*. demonstrated that *Brucella abortus* subvert the autophagy machinery of host cells to establish an intracellular niche favorable for its replication [61]. Regulation of autophagy pathway is repressed as indicated by a Bayesian *z*-score <−2.24 at 15 and 30 min p.i. (**Table 2**) which supports *Brucella*'*s* ability of taking advantage of the repressed autophagy machinery for its own survival instead of being engulfed and killed.

Pathways related to the immune system i.e. Leukocyte transendothelial migration, Toll-like receptor signaling, Hematopoietic cell lineage, Fc-epsilon RI signaling, B cell receptor signaling, B cell receptor complex, T cell receptor signaling, Apoptotic signaling in response to DNA damage, Antigen processing and presentation, JAK-STAT signaling and Natural killer cell mediated cytotoxicity were activated as indicated by a Bayesian *z*-score of >2.24 across all the time points (**Table 2**). The strong activation of these innate immune response pathways should suggest that the host is mounting an appropriate protective immune response. However, *Brucella* are known to successfully evade the host's response suggesting that *Brucella* may be selectively subverting critical signaling mechanisms. For instance, expression of the two key innate immunity anti-*Brucella* cytokines [62] such as TNF-α and IL12p40 (or IL12B) were not significantly changed (Bayesian *z*-score of >|2.24|) throughout the study. These results are in agreement with previous publications that had identified several *Brucella*'*s* strategies to avoid activation of innate immune system during the onset of the infection [39], [41], [63], [64], [65]. As examples, the Toll-Like Receptor and Cytokine-cytokine Receptor Interaction pathways were examined in more detail to determine if any important signaling events were disrupted following *Brucella* invasion as described next.

**Figure 7:**
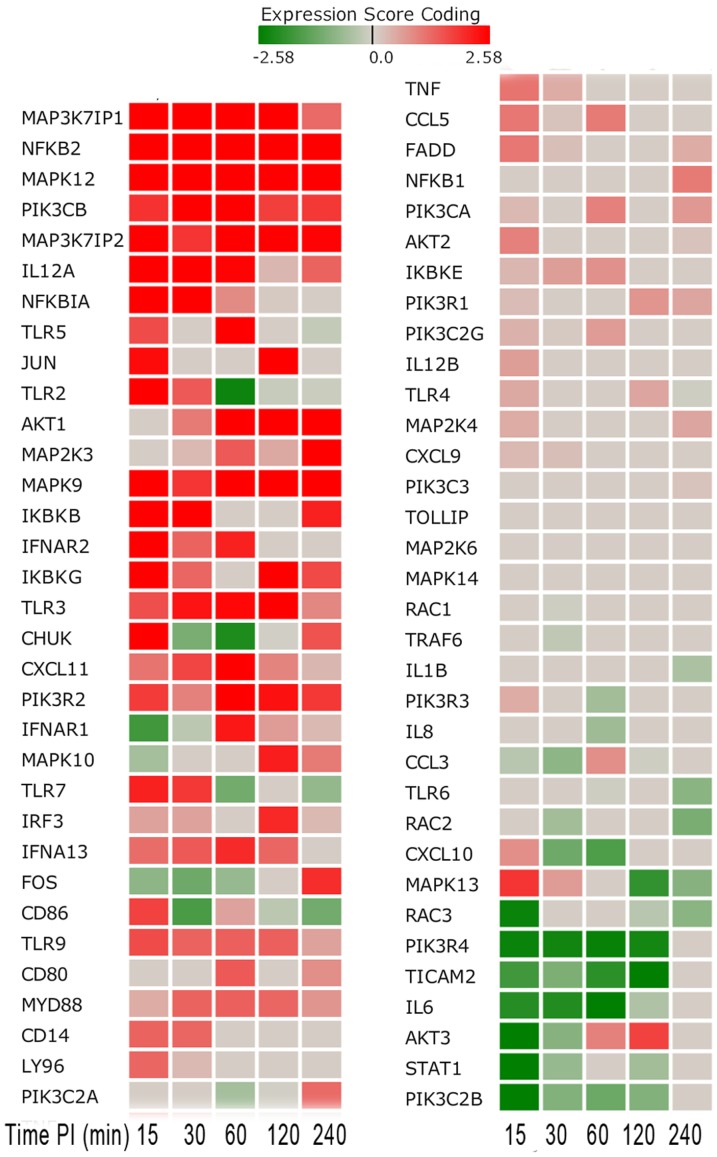
Toll-like Receptor Related Gene Score Heat Map by Time Point Post-Inoculation. The heat map shows a number of up-regulated genes occurring in the early stage (15–60 minutes post-inoculation) as indicated by the darker red boxes as well as down-regulated genes as indicated by the darker green boxes.

Toll-like receptor signaling (TLRS) pathway subversion. TLRs are crucial components of the innate immune system that recognize conserved microbial components and trigger antimicrobial responses. With the triggering of the TLRS pathway, it could be presumed that the host had initiated an effective immune response. Examining this pathway at the network and gene expression level indicated that the source of pathway perturbation comes from genes that are both highly up-regulated and interestingly, a number of repressed genes over the complete time course. **Figure 7** shows the Bayesian *z*-score gene expression heat map for all genes on the TLRS pathway, and **Table 5** shows z-scores of those genes significantly expressed in the pathway. Our analysis reveals that the only toll-like receptors differentially expressed during the study, were *TLR2* (activated at 15 min p.i. and repressed at 1 h p.i.), *TLR3* (activated at 0.5, 1 and 2 h p.i.) and *TLR5* (significantly up-regulated only at 15 min p.i.), but not for any other toll-like receptors, which is consistent with the hypothesis that *Brucella* modify, reduce and/or hide PAMP-bearing molecules to reach its replication niche before host immune system's detection [39]. In addition, *Brucella* protein Btp1 (recently re-named BtpA) and BtpB interfere with TLR signaling by producing inhibitory homologues of the TIR (Toll/Interleukin 1 receptor) domain that block MyD88-induced activation of *NFkβ* or *NFkβ* nuclear translocation [14], [66], [67]. The persistent inhibition of *NFkβ1* has been linked to inappropriate immune cell development and continues to provide evidence that supports the lack of inflammatory response through subversion of key signaling events in the TLRS pathway. Furthermore, the toll-like receptor signaling appears defective in that it is not producing the expected expression pattern for proinflammatory cytokines. For instance, proinflammatory cytokines-encoded genes such as *IL-1β*, *TNF-α*, *IL-6* and *IL8* are either not differentially expressed or are down-regulated. Additionally, chemokines *CCL3* (MIP-1α), *CCL5* (RANTES), *CXCL9*, *CXCL10*, and *CXCL11* -encoded genes were not significantly expressed and suggesting a potential disruption of monocyte and natural killer cell stimulation and T-cell migration. This lack of expression of inflammatory mediators may partially explain the absence of morphologically detectable inflammation at the site of infection [5].

**Table 5:** Genes Significantly Expressed in the TLRS Pathway (*z*-score |2.24|).

Symbol	Description	0.25	0.5	1 h	2 h	4 h
MAP3K7IP1	mitogen-activated protein kinase kinase kinase 7 interacting protein 1	3.15	4.67	5.83	5.27	1.28
NFKB2	nuclear factor of kappa light polypeptide gene enhancer in B-cells 2 (p49/p100)	3.46	3.92	5.44	3.83	3.04
MAPK12	mitogen-activated protein kinase 12	3.6	4.28	3.78	4.67	5.32
PIK3CB	phosphoinositide-3-kinase, catalytic, beta polypeptide	1.96	2.6	4.86	1.81	1.89
MAP3K7IP2	mitogen-activated protein kinase kinase kinase 7 interacting protein 2	3.3	1.92	4.32	3.79	2.52
IL12A	interleukin 12A (natural killer cell stimulatory factor 1, cytotoxic lymphocyte maturation factor 1,	3.5	3	2.51	0.34	1.37
NFKBIA	nuclear factor of kappa light polypeptide gene enhancer in B-cells inhibitor, alpha	3.28	2.59	0.88	0.09	0
TLR5	toll-like receptor 5	1.64	0	3.23	0	−0.21
JUN	jun oncogene	2.42	0	0	3.22	0
TLR2	toll-like receptor 2	3.17	1.48	−2.41	−0.17	−0.15
AKT1	v-akt murine thymoma viral oncogene homolog 1	0	1.05	3.14	3.15	2.82
MAP2K3	mitogen-activated protein kinase kinase 3	0	0.29	1.48	0.49	3.09
MAPK9	mitogen-activated protein kinase 9	3.05	1.9	3.08	2.95	2.68
IKBKB	inhibitor of kappa light polypeptide gene enhancer in B-cells, kinase beta	2.96	2.55	0	0	2.17
IFNAR2	interferon (alpha, beta and omega) receptor 2	2.92	1.36	2.17	0	0
IKBKG	inhibitor of kappa light polypeptide gene enhancer in B-cells, kinase gamma	2.69	1.31	0.04	2.84	1.69
TLR3	toll-like receptor 3	1.59	2.33	2.46	2.78	0.89
CHUK	conserved helix-loop-helix ubiquitous kinase	2.77	−1.12	−2.21	−0.02	1.54
CXCL11	chemokine (C-X-C motif) ligand 11	1.16	1.7	2.67	0.94	0.34
PIK3R2	phosphoinositide-3-kinase, regulatory subunit 2 (beta)	1.82	0.99	2.59	2.34	1.86
IFNAR1	interferon (alpha, beta and omega) receptor 1	−1.79	−0.31	2.26	0.68	0.32
RAC3	ras-related C3 botulinum toxin substrate 3 (rho family, small GTP binding protein Rac3)	−2.42	0	0	−0.34	−0.91
PIK3R4	phosphoinositide-3-kinase, regulatory subunit 4	−2.44	−2.34	−2.48	−2.3	0
TICAM2	toll-like receptor adaptor molecule 2	−1.79	−1.05	−2.05	−2.55	0
IL6	interleukin 6 (interferon, beta 2)	−2.12	−2.16	−2.58	−0.48	0
AKT3	v-akt murine thymoma viral oncogene homolog 3 (protein kinase B, gamma)	−2.88	−0.93	0.97	1.76	0
STAT1	signal transducer and activator of transcription 1, 91 kDa	−3.04	−0.75	0	−0.61	0
PIK3C2B	phosphoinositide-3-kinase, class 2, beta polypeptide	−3.5	−0.98	−1.27	−0.99	0

The table lists significantly perturbed (activated or repressed) genes at any time among the 5 different time points (0.25 h through 4 h p.i.).

#### Cytokine-cytokine receptor interactions (CCRI) pathway subversion

**Figure 8:**
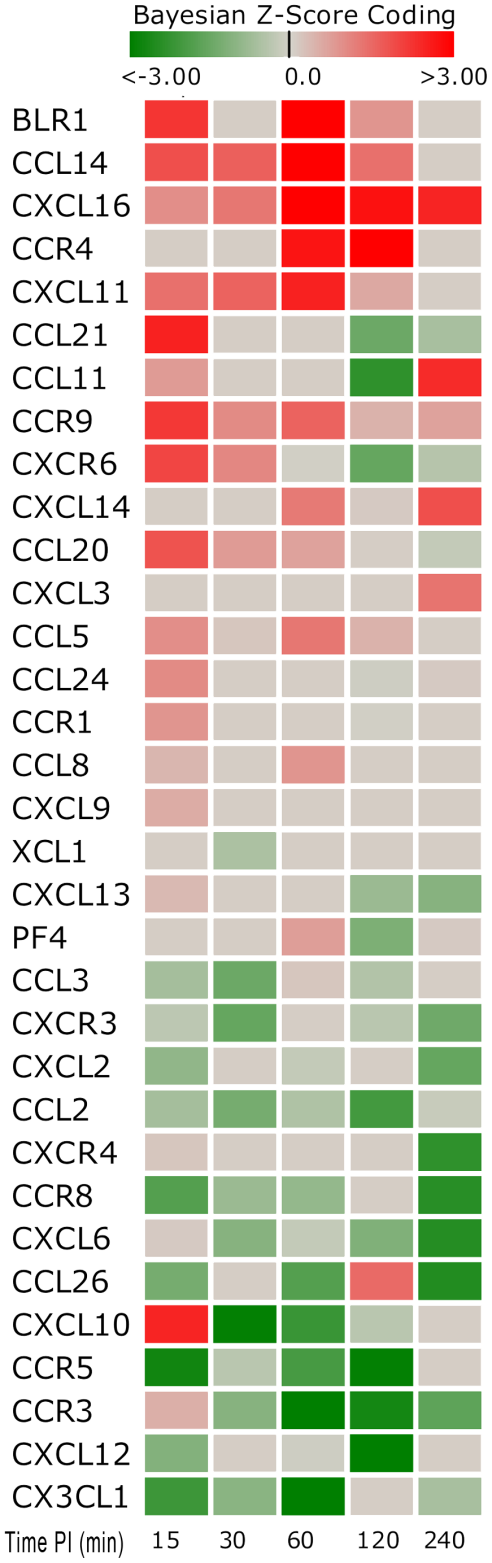
Chemokine Gene Score Heat Map by Time Point Post-Inoculation. The heat map shows a number of both up-regulated and down-regulated genes (15–240 minutes post-inoculation). Note that there were large numbers of down-regulated and non-expressed chemokine genes. Red indicates an activated state while green indicates repression and grey is no expression change from control.

The CCRI pathway was activated in the early stage of infection with several genes dominating the activation. These genes were involved in extracellular membrane receptor interaction that included chemokines (CC and CXC), interleukins (ILs) and platelet-derived growth factors (PDGFs). Chemokines and their receptors are important for the migration of various cell types into inflammatory sites. With regard to the chemokines, only *CCL21*, *CXCL10*, and *BLR1* were highly up-regulated at 15 min p.i.; *BLR1*, *CCL14*, *CXCL16*, *CXCL11* and *CCR4* were significantly expressed at 1 h p.i.; and only *CCL11* and *CXCL16* became significantly up-regulated at 4 h p.i. The other remaining chemokines were either down-regulated or minimally expressed as shown in **Figure 8**.

**Figure 9:**
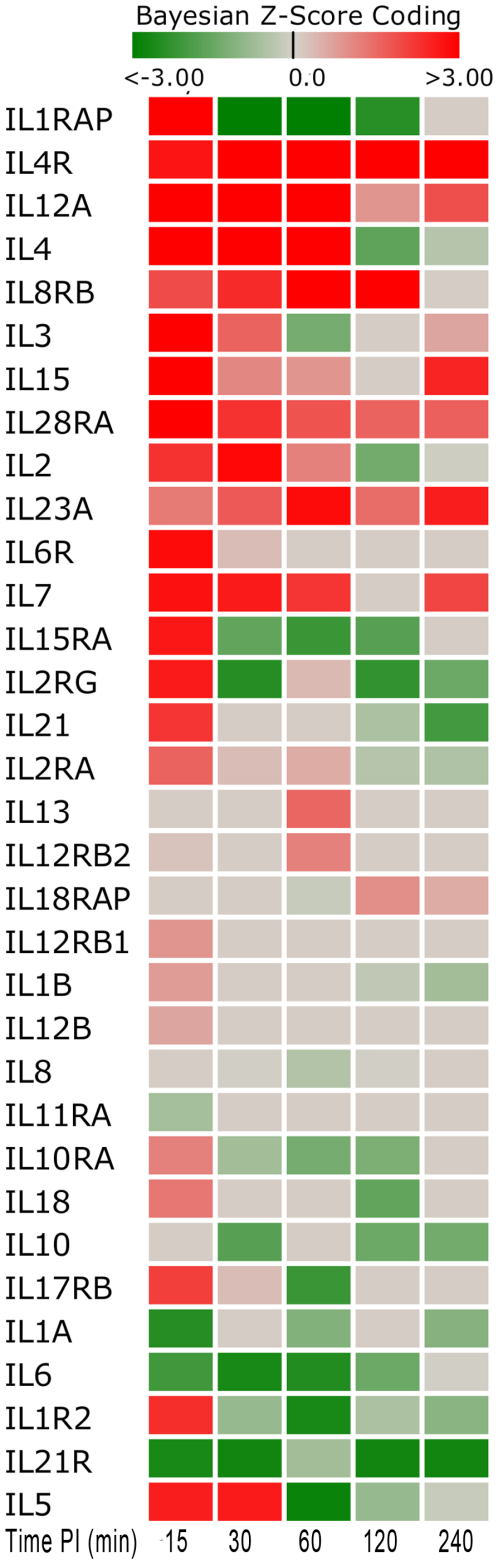
Interleukin Gene Score Heat Map by Time Point Post Inoculation. The heat map shows a number of both up-regulated and down-regulated genes (15–240 minutes post-inoculation). The heat map shows that much of the up-regulation is short-lived for many of these genes and reversed expression direction or minimally expressed in later time points. Red indicates an activated state while green indicates repression and grey is no expression change from control.

The function of the immune system depends in a large part on interleukins (ILs) that are predominately synthesized by helper CD4+ T lymphocytes, as well as through monocytes, macrophages, and endothelial cells. Interleukins promote the development and differentiation of T, B and hematopoietic cells. The strongly expressed genes encoded by interleukins or their receptors at 15 min post-*Brucella* infection include *IL-1RAP*, *IL-12A, IL-4, IL-3, IL-15, IL-28RA, IL-6R, IL-7, IL-15RA, IL-2RG, IL-5, IL-2,* and *IL-10RB.* However, the up-regulation was short-lived since many of these genes reversed direction of expression or become minimally expressed in later time points as shown in **Figure 9**. Their biological roles are summarized in Supplemental Table 28 (**Table S28 in File S1**). *IL-1RAP*, *IL-2RG*, *IL-10RB*, *IL-15RA* reversed expression at 30 min p.i. and later became significantly down-regulated. *IL-1RAP* induces synthesis of acute phase and proinflammatory proteins during infection or tissue damage; the *IL-2RG* gene is an important signaling component of many interleukin receptors, including those of interleukin −2, −4, −7 and −21; the gene *IL-10RB* encodes a cell surface receptor required for the activation of five cytokines: IL10, IL22, IL26, IL28 and IL29; and *IL-15RA* encodes a receptor that is reported to enhance cell proliferation and expression of apoptosis inhibitor BCL2L1/BCL2-XL and BCL2. This down-regulation of such genes at 30 min p.i. suggested that *Brucella* manipulate the host's immune response for survival and proliferation after its initial invasion. Interestingly, the soluble epithelial factors, *IL-7* and *IL-15*, maintained higher expression levels from 15 to 60 min p.i. The proteins encoded by these genes differentially regulate homeostasis of intraepithelial lymphocytes and other mucosal leukocytes. *IL7* can be produced locally by intestinal epithelial and epithelial goblet cells, and may serve as a regulatory factor for intestinal mucosal lymphocytes.

The elevated expression of platelet-derived growth factors (*PDGF*) has been linked to early signaling events for infection by intracellular pathogens. Only two *PDGF* pathway genes, *VEGFB* and *CSF1* were strongly up-regulated at some time points in the early stage (15–60 min p.i.). *VEGFB* signals via the endothelial receptor *FLT1* which encodes a receptor tyrosine-kinase that plays a key role in vascular development and regulation of vascular permeability. However, *FLT1* was strongly down-regulated throughout the time course. *CSF1* encodes a cytokine that plays an essential role in the regulation of survival, proliferation and differentiation of hematopoietic precursor cells, especially mononuclear phagocytes, such as macrophages and monocytes, the *in vivo* refuge of *Brucella*. The *KIT* gene was strongly down-regulated across all time points. *KIT* encodes a tyrosine-protein kinase that acts as cell-surface receptor for the cytokine KITLG/SCF and plays an essential role in the regulation of cell survival and proliferation, hematopoiesis, stem cell maintenance, gametogenesis, mast cell development, migration and function, and in melanogenesis. The down regulation of KIT may lead to the disruption of a number of important signaling events and may be a novel survival/proliferation mechanism exploited by *Brucella*.

## Conclusions

In summary, our systems biology pathway and GO analyses of the *in vivo* host intestinal transcriptome revealed that in the early phase of infection, *B. melitensis* actively modulated host responses to avert pathological lesions and immune-based inflammatory cellular pathways to rapidly invade Peyer's patch, metastasize to mesenteric lymph nodes and quickly establish bacteremia. As expected, the analysis identified genes and pathways previously known to have roles in *Brucella* infection and also detected new infection-related genes, pathways and GO terms. Molecular analysis provided evidence that the enteric mucosal barrier is compromised during early time post-infection. We identified cell molecule adhesion (CAM) and ECM receptor interaction pathways that were perturbed, more specifically *SDC2, ITGAL, ITGB2,* and *IBSP* genes. Additionally, several of the pathways of the enteric Tight Junction of the mucosa were significantly repressed in the early stages of infection, especially *NRAS*, *SPTAN1*, *PRKCG*, *PPP2R2A*, *EPB41*, *PTEN*, *CSNK2B*, *YES1*, *RHOA*, *CSNK2A1*, *MYL5*, *CGN*, *CLDN1*, *CDC42*, and *AKT3* genes. Likewise, the Trefoil Factor Initiated Mucosal Healing pathways were significantly suppressed in early stage of infection, more specifically, *PTK2*, *GHR*, *RHOA*, *CTNNB1, MAPK3, and* SHC1 genes. Our data confirmed prior observations that Toll-Like Receptor Signaling pathways were largely subverted, apparently by *Brucella* reducing or hiding PAMP-bearing molecules to reach its niche before host immune detection. While the Cytokine-Cytokine Receptor Interactions (CCRI) pathways were initially up-regulated very early in the infection, this response was very short-lived and quickly down-regulated within the first hour post-infection. In summary, our data indicate that the pathogenesis of the early infectious process of *B. melitensis* consists of compromising the mucosal immune barrier and subverting critical immune response mechanisms.

## Supporting Information

File S1 **Supplemental figures and tables. Figure A in File S1. Validation of Bovine Microarray Results by Quantitative Real Time-PCR.** cDNA was synthesized from the same RNA samples used for microarray hybridization. Five randomly selected genes (A  =  BPI; B  =  MAPK1; C  =  MIF; D  =  CCL2; E  =  IL8.) that were differentially expressed by microarrays in B. melitensis-infected bovine Peyer's patch between 15 min and 4 h p.i. as compared to non-infected tissues (control) extracted at the same time points, were validated by quantitative RT-PCR. Fold changes was normalized to the expression of GAPDH and calculated using the ΔΔCt method. All tested genes at all time points had fold-changes altered in the same direction in microarray and qRT-PCR. White bars represent fold-change by microarray analysis and black bars represent fold-change by qRT-PCR. **Table S1 in File S1. Detailed List of Host Genes with Differential Expression** (z-score >|2.24|) **in B. melitensis Infected vs. Control Bovine Jejunal-Ileal Peyer's Patch in at least one time point.** Black numbers in the body of the table indicate differentially expressed (activated: (+) numbers; repressed: (−) numbers) while red numbers represent non-differentially expressed genes. **Table S2 in File S1. Bayesian z-score for All Host Pathways in B. melitensis Infected vs. Control Bovine Jejunal-Ileal Peyer's Patch.** Black numbers in the body of the table indicate differentially expressed (activated: (+) numbers; repressed: (−) numbers) while red numbers represent non-differentially expressed genes. **Table S3 in File S1. List of All Biological Process-Related Host Genes Differentially Expressed in B. melitensis Infected vs. Control Bovine Jejunal-Ileal Peyer's Patch.** Black numbers in the body of the table indicate differentially expressed (activated: (+) numbers; repressed: (−) numbers) while red numbers represent non-differentially expressed genes. **Table S4 in File S1. List of All Cellular Component-Related Host Genes Differentially Expressed in B. melitensis Infected vs. Control Bovine Jejunal-Ileal Peyer's Patch.** Black numbers in the body of the table indicate differentially expressed (activated: (+) numbers; repressed: (−) numbers) while red numbers represent non-differentially expressed genes. **Table S5 in File S1. List of All Molecular Function-Related Host Genes Differentially Expressed in B. melitensis Infected vs. Control Bovine Jejunal-Ileal Peyer's Patch.** Black numbers in the body of the table indicate differentially expressed (activated: (+) numbers; repressed: (−) numbers) while red numbers represent non-differentially expressed genes. **Table S6 in File S1. Group 1 - GO Biological Process Terms Significantly Perturbed Early (Activated or Repressed) but Not Later.** Black numbers in the body of the table indicate differentially expressed (activated: (+) numbers; repressed: (−) numbers) while red numbers represent non-differentially expressed genes. **Table S7 in File S1. Group 2 - GO Biological Process Terms Consistently Perturbed (Activated or Repressed) Throughout the Experiment; Not Significantly Perturbed Early but Later; or Consistently Altered Perturbation (Activated to Repressed or Vice Versa).** Black numbers in the body of the table indicate differentially expressed (activated: (+) numbers; repressed: (−) numbers) while red numbers represent non-differentially expressed genes. **Table S8 in File S1. Group 1 - GO Cellular Components Terms Significantly Perturbed Early (Activated or Repressed) but Not Later.** Black numbers in the body of the table indicate differentially expressed (activated: (+) numbers; repressed: (−) numbers) while red numbers represent non-differentially expressed genes. **Table S9 in File S1. Group 2 - GO Cellular Component Terms Consistently Perturbed (Activated or Repressed) Throughout the Experiment; or Terms Not Significantly Perturbed Early but Later; or Consistently Altered Perturbation (Activated to Repressed or Vice Versa).** Black numbers in the body of the table indicate differentially expressed (activated: (+) numbers; repressed: (−) numbers) while red numbers represent non-differentially expressed genes. **Table S10 in File S1. Group 1 - GO Molecular Function Terms Significantly Perturbed Early (Activated or Repressed) but Not Later.** Black numbers in the body of the table indicate differentially expressed (activated: (+) numbers; repressed: (−) numbers) while red numbers represent non-differentially expressed genes. **Table S11 in File S1. Group 2 - GO Molecular Function Terms Consistently Perturbed (Activated or Repressed) Throughout the Experiment; or Terms Not Significantly Perturbed Early but Later; or Consistently Altered Perturbation (Activated to Repressed or Vice Versa).** Black numbers in the body of the table indicate differentially expressed (activated: (+) numbers; repressed: (−) numbers) while red numbers represent non-differentially expressed genes. **Table S12 in File S1. Comparative Table of Group 1 and Group 2 Gene Ontology Terms Clustered to the Top 15 Primary Immune Related Biological Process Categories.** This table shows lists the mapping of perturbed GO term gene groups to their higher level GO category definitions. The number of perturbed GO terms that mapped to each category is provided in the Count column. **Table S13 in File S1. Group 1 - Detailed Listing of the Top 15 Highly Perturbed Gene Ontology Terms Mapped to Major Immune Related Biological Process.** This table lists each GO term that was mapped (clustered) to a main category related to an immune response. There were 108 main categories to which 686 perturbed GO term sets of genes resulted in mapping to 55 categories. Group 1 represents those GO terms that were significantly perturbed at earlier time points (either activated or repressed) but not at later time points. **Table S14 in File S1. Group 2 - Detailed Listing of the Top 15 Highly Perturbed Gene Ontology Terms Mapped to Major Immune Related Biological Process.** This table lists each GO term that was mapped (clustered) to a main category related to an immune response. There were 108 main categories to which 114 perturbed GO term sets of genes resulted in mapping to 23 categories. Group 2 are GO terms consistently perturbed (up- or down-regulated) throughout the experiment; or GO terms not significantly perturbed at earlier time points but at later times; or GO terms that consistently changed their state of perturbation (from activated- to repressed or vice versa). **Table S15 in File S1. Comparative Table of Group 1 and Group 2 Gene Ontology Terms Clustered to Primary Cellular Component Categories.** The mapping of terms in this table is to major categories related to the cellular component categories. **Table S16 in File S1. Group 1 - Detailed Listing of Highly Perturbed Gene Ontology Terms Mapped to Major Cellular Component Categories.** This table lists each GO term that was mapped (clustered) to a main category related to its cellular component association. There were 12 main categories to which 112 perturbed GO term sets of genes resulted in mapping to 9 categories. Definition of Group 1 is same as described in Table S13. **Table S17 in File S1. Group 2 - Detailed Listing of Highly Perturbed Gene Ontology Terms Mapped to Major Cellular Component Categories.** This table lists each GO term that was mapped (clustered) to a main category related to its cellular component association. There were 12 main categories to which 26 perturbed GO term sets of genes resulted in mapping to 6 categories. Definition of Group 2 is same as described in Table S14. **Table S18 in File S1. Comparative Table of Group 1 and Group 2 Gene Ontology Terms Clustered to Primary Molecular Function Categories.** The mapping of terms in this table is to major categories related to the primary molecular function categories. **Table S19 in File S1. Group 1 - Detailed Listing of Highly Perturbed Gene Ontology Terms Mapped to Major Molecular Function Categories.** This table lists each GO term that was mapped (clustered) to a main category related to its cellular component association. There were 17 main categories to which 220 perturbed GO term sets of genes resultedin mapping to 10 categories. Definition of Group 1 is same as described in Table S13. **Table S20 in File S1. Group 2 - Detailed Listing of Highly Perturbed Gene Ontology Terms Mapped to Major Molecular Function Categories.** This table lists each GO term that was mapped (clustered) to a main category related to its cellular component association. There were 17 main categories to which 38 perturbed GO term sets of genes resulted in mapping to 6 categories. Definition of Group 1 is same as described in Table S14. **Table S21 in File S1. Mechanistic Genes with Overlapping Intersections among Multiple Pathways.** This table lists 476 unique mechanistic genes associated with the list of pathways in Table 2. The table shows the number of pathways for which each gene has an intersection. The Bayesian z-score by time point is provided for each gene. **Table S22 in File S1. Significantly Down-Regulated Mechanistic Genes with Bayesian z-score <−2.24 in the Early Stage of Infection (15, 30, & 60 minutes post-infection).** The table lists significantly perturbed down-regulated genes with a high degree of overlap (intersection or cross-talk) across multiple pathways (5 or more intersections) associated with those pathways listed in Table 2. Significant down-regulated Bayesian z-scores in the first hour p.i. are bolded. **Table S23 in File S1. Significantly Up-Regulated Mechanistic Genes with Bayesian z-score >2.24 in the Early Stage of Infection (15, 30, & 60 minutes post-infection).** The table lists significantly perturbed up-regulated genes with a high degree of overlap (intersection or crosstalk) across multiple pathways (5 or more intersections) associated with those pathways listed in Table 2. Significant up-regulated Bayesian z-scores in the first hour p.i. are **bolded**. **Table S24 in File S1. Description of the Biological Role of Dominant Down-Regulated Mechanistic Genes in Tight Junction Pathway. Table S25 in File S1. Description of the Biological Role of Dominant Down-Regulated Mechanistic genes in Trefoil Factors Initiated Mucosal Healing. Table S26 in File S1. Significant Perturbed Genes and Their Biological Roles for the Lectin Pathway. Table S27 in File S1. Significant Perturbed Genes and Their Biological Roles for Phagocytosis Gene Ontology Group. Table S28 in File S1. Interleukin Mechanistic Genes Significantly Perturbed at 15 minutes post-infection.**
(ZIP)
