# Supplementary material for: Systems Biology Analysis of Brucella Infected Peyer's Patch Reveals Rapid Invasion with Modest Transient Perturbations of the Host Transcriptome
Source: PLoS One. 2013 Dec 9;8(12):e81719. doi: 10.1371/journal.pone.0081719 (PMC3857238; doi:10.1371/journal.pone.0081719)
Supplement: File S1 — Supplemental figures and tables. Figure A in File S1. Validation of Bovine Microarray Results by Quantitative Real Time-PCR. cDNA was synthesized from the same RNA samples used for microarray hybridization. Five randomly selected genes (A = BPI; B = MAPK1; C = MIF; D = CCL2; E = IL8.) that were differentially expressed by microarrays in B. melitensis-infected bovine Peyer's patch between 15 min and 4 h p.i. as compared to non-infected tissues (control) extracted at the same time points, were validated by quantitative RT-PCR. Fold changes was normalized to the expression of GAPDH and calculated using the ΔΔCt method. All tested genes at all time points had fold-changes altered in the same direction in microarray and qRT-PCR. White bars represent fold-change by microarray analysis and black bars represent fold-change by qRT-PCR. Table S1 in File S1. Detailed List of Host Genes with Differential Expression (z-score >|2.24|) in B. melitensis Infected vs. Control Bovine Jejunal-Ileal Peyer's Patch in at least one time point. Black numbers in the body of the table indicate differentially expressed (activated: (+) numbers; repressed: (−) numbers) while red numbers represent non-differentially expressed genes. Table S2 in File S1. Bayesian z-score for All Host Pathways in B. melitensis Infected vs. Control Bovine Jejunal-Ileal Peyer's Patch. Black numbers in the body of the table indicate differentially expressed (activated: (+) numbers; repressed: (−) numbers) while red numbers represent non-differentially expressed genes. Table S3 in File S1. List of All Biological Process-Related Host Genes Differentially Expressed in B. melitensis Infected vs. Control Bovine Jejunal-Ileal Peyer's Patch. Black numbers in the body of the table indicate differentially expressed (activated: (+) numbers; repressed: (−) numbers) while red numbers represent non-differentially expressed genes. Table S4 in File S1. List of All Cellular Component-Related Host Genes Differentially Expressed in B. [file pone.0081719.s001.zip › MS Bmel Final Suppl File Figure Tables 31x2013/Supplementary File S1.docx]

**Supplementary File S1**

**The quality of samples and reliability of bovine microarray data**

Bioanalysis determined that RNA from the experimental samples and the reference RNA were of good to excellent quality (RIN > 7.0, 28S/18S ratio > 1.4, OD_260/280_ > 2.0, OD_260/230_ > 1.8 for experimental samples; and RIN = 9.7, 28S/18S ratio = 2.1, OD_260/280_ = 2.01, OD_260/230_ = 1.85 for reference RNA). Hybridized on the arrays, the reference bovine RNA generated readable signal intensities on more than 85% of the spots on the microarray (SNR > 3SD above background). No slides (or individual arrays) were removed, as all R^2^ values were above acceptable levels (0.70 or greater) after normalization and filtering.

To determine the alterations in gene expression, microarray data were analyzed by a Bayesian inference analysis model. The *z*-score > |2.24| reflects 97.5% confidence in the data and was used as the threshold for inference testing. Bayesian inference identified a progressive host gene expression modification with the highest activity at one hour p.i. in infected intestinal loops tissues compared with control tissues, which decreased at later time points (**Figure 2**). A total of 2,916 different genes (2,286 up- vs. 630 down-regulated) were differential expressed (DE) in the first 4 h p.i. compared to control loops. The data from the time points indicated that 1,587 genes (1,088 up- and 499 down-regulated) were DE at only one time point, 730 (612 up- and 118 down-regulated) at two consecutive time points and 372 (359 up- vs. 13 down-regulated) and 192 genes (188 up- vs. 4 down-regulated) were DE at 3 and 4 consecutive time points, respectively. There were only 37 ORF (all up-regulated) with DE at all five time points, and less than 20 genes with DE at two non-consecutive time points (**Table S1 in File S1**). At every time point, the number of bovine genes up-regulated was always higher than the down-regulated ones (**Figure 2**).

Microarray gene expression data were validated by qRT-PCR. Five bovine genes, encoded for immunity-related products and determined to be significantly affected in the first 4 h p.i., were chosen for verification at every time point (i.e. 30 data points). As shown by the representative examples in Supplemental Figure A (**Fig. A in File S1),** gene expression changes were consistent between microarray and qRT-PCR for genes with increased expression or genes with decreased expression relative to the negative control.

**Dynamic Bayesian network modeling analysis of pathways and GO terms activation revealed an early perturbed metabolic state that rapidly returned to normal levels**

To better understand the complex molecular interactions between host and pathogen, gene expression data were mapped to 220 molecular interaction pathways and 3,225 GO terms using the Dynamic Bayesian Gene Group Activation (DBGGA) method described more fully elsewhere (Adams *et al*., 2011, Adams *et al*., 2011, Khare *et al*., 2012, Lawhon *et al*., 2011). DBGGA creates a Dynamic Bayesian network (DBN) model for each pathway based on KEGG (KEGG webpage: [www.kegg.com](http://www.kegg.com); Loor *et al*., 2007, Everts *et al*., 2005, Kanehisa *et al*., 2000, KEGG Kyoto Encyclopedia). The pathway and GO DBN models were trained with the control group data (uninfected) and the experimental data (infected) used as evidence to test how different experimental data are applied in fitting the control model. This difference is determined by measuring the negative log-likelihood that, in turn, was transformed to a *z*-score test statistic that is referred to, hereafter, as the Bayesian *z*-score. This method ranked pathway and GO terms at each time point to determine differences between experimental conditions (Lawson *et al*., 2011, Adams *et al*., 2011). Similarly, how well the results of individual genes fit a model was also determined, producing Bayesian *z*-scores for each gene within a given pathway or GO category. The DBGGA method employed a Bayesian variance estimator to infer a better prediction of the variances for the genes that have a low number of biological replicates (Baldi *et al*., 2001, Long *et al*., 2001). The Dynamic Bayesian network modeling analysis identified 207 pathways that were perturbed (Bayesian *z*-score >|2.24|) between inoculated and control loops at least in one time point throughout the experiment, and 13 pathways that had never been activated or repressed during the course of the experiment (**Table S2 in File S1**). A global graphical representation of the number of pathways significantly altered at every time point showed a higher expression during the first hour p.i. (199 pathways at 0.25h, 176 at 0.5h, 194 at 1h) that decreased at the later time points (181 pathways at 2h, and 83 at 4h p.i.). There was always a higher number of activated than repressed pathways at every time point.

Focusing our analysis on pathways involved in response to *Brucella* spp. infection has enabled us to follow the behavior of Cell communications, Cell growth and death, Cell motility, Immune system, Infectious disease, Membrane transport, Signal transduction and Signaling molecules and interaction categories. These categories create a subgroup of 56 pathways. The analysis of this subgroup revealed that 53 were differentially expressed at 15 min (47 activated and 6 repressed), 52 were altered at 1 h p.i. (47 activated and 5 repressed), 49 (41 activated and 8 repressed) at 2 h, and then declined to 25 (24 activated and 1 repressed) altered pathways at 4 h p.i. (**Table 2 in File S1**).

Also a DBGGA running over 3,225 gene ontology (GO) terms identified a higher number of terms perturbed at earlier more than at later time points. A graphical representation of perturbed GO terms during the experiment revealed a similar image observed for altered pathways, with a higher number of GO terms expressed at 15 min that decreased at later time points (**Figure 3, Table S3, S4, S5 in File S1**). Simultaneously, 2 expression groups were identified among the 3,225 GO terms analyzed: Group 1 - GO terms that were significantly perturbed at earlier time points (either activated or repressed) but not at later time points; and Group 2 - GO terms consistently perturbed (activated or repressed) throughout the experiment; GO terms not significantly perturbed at earlier time points but at later times; or GO terms that consistently changed their state of perturbation (from activated to repressed or vice versa). GO terms in the first group could be considered part of the host response to eliminate the pathogen that is averted by *Brucella*, while those in the second group are considered GO terms that *Brucella* may need to regulate (differentially activate or repress) during the infection process. The analysis of the GO *biological process* terms revealed that there were 686 terms that belonged to Group 1 and 114 to Group 2 (**Table S6, S7 in File S1**). Among the GO *cellular components* terms, the analysis showed 123 terms belonged to Group 1 and 26 to Group 2 (**Table S8, S9 in File S1**); and 220 GO *molecular functions* terms belonged to Group 1 and 38 to Group 2 (**Table S10, S11 in File S1**).

Further clustering of the GO terms for Group 1 and Group 2 to immune system related biological processes terms was conducted to help determine the primary differences between the two groups. The immune related GO ontologies are a specialized selection of ontologies consisting of 110 GO terms representing a customized subset of terms similar to the Gene Ontology Consortium [GO Slim and Subset Guide <http://www.geneontology.org/GO.slims.shtml>]. This subset of terms map to major immune related ontology categories that give a broad overview of the ontology content without the detail of the specific fine grained terms. **Table S12 in File S1**shows the comparison of the top 15 major categories for both Groups 1 and 2. For Group 1 there were 686 enriched detailed terms that mapped to 55 of the 110 major immune categories, while for Group 2 there were only 114 detailed terms that mapped to 23 of the 110 major categories. Full details of the GO term mappings underlying **Table S12** **in File S1** is provided in **Table S13** and **S14 in File S1**. Likewise, clustering of GO terms for Group 1 and 2 was completed for a customized subset of major cellular component categories as shown in **Table S15 in File S1**. For Group 1, there were 123 perturbed detailed cellular component terms that mapped to 9 major categories, while for Group 2, there were only 26 enriched detailed terms that mapped to 6 major cellular component categories. Full details of the GO term mapping for Table 4 are provided in **Table S16 and S17 in File S1**. GO clustering for Group 1 and 2 to a customized subset of major molecular function categories produced the results in **Table S18 in File S1**. For Group 1, there were 220 detailed enriched GO terms that mapped to 10 major molecular function categories, while for Group 2 there were 38 detailed enriched terms that mapped to 6 major molecular function categories. Full details of the GO term mapping for Table 5 are provided in **Table S19** and **S20 in File S1**.

Overall, these results indicate that host metabolic and signaling pathways and GO terms are pronouncedly perturbed at a very early time post-*Brucella* infection, with a strong tendency to rapidly return to a normal state at later time points.

**Dynamic Bayesian modeling analysis of bovine microarray reveals biosignature candidate genes**

To explore further differences between inoculated and control groups, deeper Bayesian analysis identified 554 unique mechanistic gene candidates in a subcategory of 37 pathways highly perturbed at earlier time points (either activated or repressed) (**Table 2**). For our analysis, a candidate mechanistic gene is identified by surpassing a Bayesian z-score > |2.24|, which implies that this gene significantly contributes to the perturbation of the overall pathway in terms of its molecular interaction and influence on other downstream genes. Of these 554 genes, 49 were identified as key intersection points having mechanistic functions in 5 or more of the 37 selected pathways and hence is assumed to have a broad regulatory (mechanistic) influence from a biological system perspective. These 554 mechanistic genes are listed in the **Table S21 in File S1**. Several interesting genes are significantly down-regulated (subverted) in the early stage (15, 30, or 60 minutes) post infection as shown in **Table S22 in File S1**, while significantly up-regulated genes are listed in **Table S23 in File S1**. The exploitation of these genes by *Brucella* are likely to be associated with its successful penetration into host cells and its evasion of the host immune response.

**References**

Adams LG, Khare S, Lawhon SD, Rossetti CA, Lewin HA, et al. (2011) Enhancing the role of veterinary vaccines reducing zoonotic diseases of humans: linking systems biology with vaccine development. Vaccine 29: 7197-7206.

Adams LG, Khare S, Lawhon SD, Rossetti CA, Lewin HA, et al. (2011) Multi-comparative systems biology analysis reveals time-course biosignatures of in vivo bovine pathway responses to B.melitensis, S.enterica Typhimurium and M.avium paratuberculosis. BMC Proc 5 Suppl 4: S6.

Khare S, Lawhon SD, Drake KL, Nunes JE, Figueiredo JF, et al. (2012) Systems Biology Analysis of Gene Expression during In Vivo Mycobacterium avium paratuberculosis Enteric Colonization Reveals Role for Immune Tolerance. PLoS One 7: e42127.

Lawhon SD, Khare S, Rossetti CA, Everts RE, Galindo CL, et al. (2011) Role of SPI-1 secreted effectors in acute bovine response to Salmonella enterica Serovar Typhimurium: a systems biology analysis approach. PLoS One 6: e26869.

Loor JJ, Everts RE, Bionaz M, Dann HM, Morin DE, et al. (2007) Nutrition-induced ketosis alters metabolic and signaling gene networks in liver of periparturient dairy cows. Physiol Genomics 32: 105-116.

Everts RE, Band MR, Liu ZL, Kumar CG, Liu L, et al. (2005) A 7872 cDNA microarray and its use in bovine functional genomics. Vet Immunol Immunopathol 105: 235-245.

Kanehisa M, Goto S (2000) KEGG: kyoto encyclopedia of genes and genomes. Nucleic Acids Res 28: 27-30.

KEGG Kyoto Encyclopedia of Genes and Genomes KL. Kyoto University and University of Tokyo, Japan.

Baldi P, Long AD (2001) A Bayesian framework for the analysis of microarray expression data: regularized t -test and statistical inferences of gene changes. Bioinformatics 17: 509-519.

Long AD, Mangalam HJ, Chan BY, Tolleri L, Hatfield GW, et al. (2001) Improved statistical inference from DNA microarray data using analysis of variance and a Bayesian statistical framework. Analysis of global gene expression in Escherichia coli K12. J Biol Chem 276: 19937-19944.
