# Supplementary material for: Systems Biology Analysis of Brucella Infected Peyer's Patch Reveals Rapid Invasion with Modest Transient Perturbations of the Host Transcriptome
Source: PLoS One. 2013 Dec 9;8(12):e81719. doi: 10.1371/journal.pone.0081719 (PMC3857238; doi:10.1371/journal.pone.0081719)
Supplement: File S1 — Supplemental figures and tables. Figure A in File S1. Validation of Bovine Microarray Results by Quantitative Real Time-PCR. cDNA was synthesized from the same RNA samples used for microarray hybridization. Five randomly selected genes (A = BPI; B = MAPK1; C = MIF; D = CCL2; E = IL8.) that were differentially expressed by microarrays in B. melitensis-infected bovine Peyer's patch between 15 min and 4 h p.i. as compared to non-infected tissues (control) extracted at the same time points, were validated by quantitative RT-PCR. Fold changes was normalized to the expression of GAPDH and calculated using the ΔΔCt method. All tested genes at all time points had fold-changes altered in the same direction in microarray and qRT-PCR. White bars represent fold-change by microarray analysis and black bars represent fold-change by qRT-PCR. Table S1 in File S1. Detailed List of Host Genes with Differential Expression (z-score >|2.24|) in B. melitensis Infected vs. Control Bovine Jejunal-Ileal Peyer's Patch in at least one time point. Black numbers in the body of the table indicate differentially expressed (activated: (+) numbers; repressed: (−) numbers) while red numbers represent non-differentially expressed genes. Table S2 in File S1. Bayesian z-score for All Host Pathways in B. melitensis Infected vs. Control Bovine Jejunal-Ileal Peyer's Patch. Black numbers in the body of the table indicate differentially expressed (activated: (+) numbers; repressed: (−) numbers) while red numbers represent non-differentially expressed genes. Table S3 in File S1. List of All Biological Process-Related Host Genes Differentially Expressed in B. melitensis Infected vs. Control Bovine Jejunal-Ileal Peyer's Patch. Black numbers in the body of the table indicate differentially expressed (activated: (+) numbers; repressed: (−) numbers) while red numbers represent non-differentially expressed genes. Table S4 in File S1. List of All Cellular Component-Related Host Genes Differentially Expressed in B. [file pone.0081719.s001.zip › MS Bmel Final Suppl File Figure Tables 31x2013/Table S26_Biological Roles Lectin Pathway mech genes.docx]

**Table S26. Significant Perturbed Genes and Their Biological Roles for the Lectin Pathway.**

| **Gene** | **Description** | **Summary** |
| --- | --- | --- |
| MASP2 | mannan-binding lectin serine peptidase 2 | The Ra-reactive factor (RARF) is a complement-dependent bactericidal factor that binds to the Ra and R2 polysaccharides expressed by certain enterobacteria. The encoded proteins are members of the trypsin family of peptidases. |
| C9 | complement component 9 | This gene encodes the final component of the complement system. It participates in the formation of the Membrane Attack Complex (MAC). The MAC assembles on bacterial membranes to form a pore, permitting disruption of bacterial membrane organization. Mutations in this gene cause component C9 deficiency. |
| C5 | complement component 5 | The protein encoded by this gene is the fifth component of complement, which plays an important role in inflammatory and cell killing processes. |
| C6 | complement component 6 | C6 is a component of complement cascade. It is part of the membrane attack complex which can insert into the cell membrane and cause cell to lyse. |
