# Supplementary material for: Systems Biology Analysis of Brucella Infected Peyer's Patch Reveals Rapid Invasion with Modest Transient Perturbations of the Host Transcriptome
Source: PLoS One. 2013 Dec 9;8(12):e81719. doi: 10.1371/journal.pone.0081719 (PMC3857238; doi:10.1371/journal.pone.0081719)
Supplement: File S1 — Supplemental figures and tables. Figure A in File S1. Validation of Bovine Microarray Results by Quantitative Real Time-PCR. cDNA was synthesized from the same RNA samples used for microarray hybridization. Five randomly selected genes (A = BPI; B = MAPK1; C = MIF; D = CCL2; E = IL8.) that were differentially expressed by microarrays in B. melitensis-infected bovine Peyer's patch between 15 min and 4 h p.i. as compared to non-infected tissues (control) extracted at the same time points, were validated by quantitative RT-PCR. Fold changes was normalized to the expression of GAPDH and calculated using the ΔΔCt method. All tested genes at all time points had fold-changes altered in the same direction in microarray and qRT-PCR. White bars represent fold-change by microarray analysis and black bars represent fold-change by qRT-PCR. Table S1 in File S1. Detailed List of Host Genes with Differential Expression (z-score >|2.24|) in B. melitensis Infected vs. Control Bovine Jejunal-Ileal Peyer's Patch in at least one time point. Black numbers in the body of the table indicate differentially expressed (activated: (+) numbers; repressed: (−) numbers) while red numbers represent non-differentially expressed genes. Table S2 in File S1. Bayesian z-score for All Host Pathways in B. melitensis Infected vs. Control Bovine Jejunal-Ileal Peyer's Patch. Black numbers in the body of the table indicate differentially expressed (activated: (+) numbers; repressed: (−) numbers) while red numbers represent non-differentially expressed genes. Table S3 in File S1. List of All Biological Process-Related Host Genes Differentially Expressed in B. melitensis Infected vs. Control Bovine Jejunal-Ileal Peyer's Patch. Black numbers in the body of the table indicate differentially expressed (activated: (+) numbers; repressed: (−) numbers) while red numbers represent non-differentially expressed genes. Table S4 in File S1. List of All Cellular Component-Related Host Genes Differentially Expressed in B. [file pone.0081719.s001.zip › MS Bmel Final Suppl File Figure Tables 31x2013/Table S27_PhagocytosisGenes.docx]

**Table S27. Significant Perturbed Genes and Their Biological Roles for Phagocytosis Gene Ontology Group.**

| **Gene** | **Description** | **Summary** |
| --- | --- | --- |
| DOCK1 | dedicator of cytokinesis 1 | This gene product binds to the SH3 domain of CRK protein. It may regulate cell surface extension and may have a role in the cell surface extension of an engulfing cell around a dying cell during apoptosis. |
| SIRPA | signal-regulatory protein alpha | The protein encoded by this gene is a member of the signal-regulatory-protein (SIRP) family, and also belongs to the immunoglobulin superfamily. SIRP family members are receptor-type transmembrane glycoproteins known to be involved in the negative regulation of receptor tyrosine kinase-coupled signaling processes. CD47 has been demonstrated to be a ligand for this receptor protein. |
| GATA2 | GATA binding protein 2 | This gene encodes a member of the GATA family of zinc-finger transcription factors that are named for the consensus nucleotide sequence they bind in the promoter regions of target genes. The encoded protein plays an essential role in regulating transcription of genes involved in the development and proliferation of hematopoietic and endocrine cell lineages. |
| AHSG | alpha-2-HS-glycoprotein | Alpha2-HS glycoprotein (AHSG), a glycoprotein present in the serum, is synthesized by hepatocytes. It is involved in several functions, such as endocytosis, brain development and the formation of bone tissue. |
| SFTPD | surfactant protein D | Contributes to the lung's defense against inhaled microorganisms. May participate in the extracellular reorganization or turnover of pulmonary surfactant. Binds strongly maltose residues and to a lesser extent other alpha-glucosyl moieties. |
| CD47 | CD47 molecule | This gene encodes a membrane protein, which is involved in the increase in intracellular calcium concentration that occurs upon cell adhesion to extracellular matrix. |
| ELMO1 | engulfment and cell motility 1 | This gene encodes a member of the engulfment and cell motility protein family. These proteins interact with dedicator of cytokinesis proteins to promote phagocytosis and cell migration. Increased expression of this gene and dedicator of cytokinesis 1 may promote glioma cell invasion. |
| SCARB1 | scavenger receptor class B, member 1 | The protein encoded by this gene is a plasma membrane receptor for high density lipoprotein cholesterol (HDL). The encoded protein mediates cholesterol transfer to and from HDL. In addition, this protein is a receptor for hepatitis C virus glycoprotein E2. |
| FCER1G | Fc fragment of IgE, high affinity I, receptor for; gamma polypeptide | The high affinity IgE receptor is a key molecule involved in allergic reactions. Regulates several aspects of the immune response. Also involved in collagen-mediated platelet activation and in neutrophil activation mediated by integrin. |
| ELMO2 | engulfment and cell motility 2 | The protein encoded by this gene interacts with the dedicator of cyto-kinesis 1 protein. Similarity to a C. elegans protein suggests that this protein may function in phagocytosis of apoptotic cells and in cell migration. |
| AZU1 | azurocidin 1 | Azurophil granules, specialized lysosomes of the neutrophil, contain at least 10 proteins implicated in the killing of microorganisms. It is also an important multifunctional inflammatory mediator. |
| CORO1A | coronin, actin binding protein, 1A | This gene encodes a member of the WD repeat protein family. Members of this family are involved in a variety of cellular processes, including cell cycle progression, signal transduction, apoptosis, and gene regulation. Alternative splicing results in multiple transcript variants. A related pseudogene has been defined on chromosome 16. |
| CDC42SE2 | CDC42 small effector 2 | Probably involved in the organization of the actin cytoskeleton by acting downstream of CDC42, inducing actin filament assembly. Alters CDC42-induced cell shape changes. In activated T-cells, may play a role in CDC42-mediated F-actin accumulation at the immunological synapse. May play a role in early contractile events in phagocytosis in  macrophages |
