# Supplementary material for: Systems Biology Analysis of Brucella Infected Peyer's Patch Reveals Rapid Invasion with Modest Transient Perturbations of the Host Transcriptome
Source: PLoS One. 2013 Dec 9;8(12):e81719. doi: 10.1371/journal.pone.0081719 (PMC3857238; doi:10.1371/journal.pone.0081719)
Supplement: File S1 — Supplemental figures and tables. Figure A in File S1. Validation of Bovine Microarray Results by Quantitative Real Time-PCR. cDNA was synthesized from the same RNA samples used for microarray hybridization. Five randomly selected genes (A = BPI; B = MAPK1; C = MIF; D = CCL2; E = IL8.) that were differentially expressed by microarrays in B. melitensis-infected bovine Peyer's patch between 15 min and 4 h p.i. as compared to non-infected tissues (control) extracted at the same time points, were validated by quantitative RT-PCR. Fold changes was normalized to the expression of GAPDH and calculated using the ΔΔCt method. All tested genes at all time points had fold-changes altered in the same direction in microarray and qRT-PCR. White bars represent fold-change by microarray analysis and black bars represent fold-change by qRT-PCR. Table S1 in File S1. Detailed List of Host Genes with Differential Expression (z-score >|2.24|) in B. melitensis Infected vs. Control Bovine Jejunal-Ileal Peyer's Patch in at least one time point. Black numbers in the body of the table indicate differentially expressed (activated: (+) numbers; repressed: (−) numbers) while red numbers represent non-differentially expressed genes. Table S2 in File S1. Bayesian z-score for All Host Pathways in B. melitensis Infected vs. Control Bovine Jejunal-Ileal Peyer's Patch. Black numbers in the body of the table indicate differentially expressed (activated: (+) numbers; repressed: (−) numbers) while red numbers represent non-differentially expressed genes. Table S3 in File S1. List of All Biological Process-Related Host Genes Differentially Expressed in B. melitensis Infected vs. Control Bovine Jejunal-Ileal Peyer's Patch. Black numbers in the body of the table indicate differentially expressed (activated: (+) numbers; repressed: (−) numbers) while red numbers represent non-differentially expressed genes. Table S4 in File S1. List of All Cellular Component-Related Host Genes Differentially Expressed in B. [file pone.0081719.s001.zip › MS Bmel Final Suppl File Figure Tables 31x2013/Table S28_interleukinMechGenes.docx]

**Table S28. Interleukin Mechanistic Genes Significantly Perturbed at 15 minutes post-infection.**

| **Down-regulated interleukins at 15 minutes p.i.** | | | | |
| --- | --- | --- | --- | --- |
| **Gene** | **Description** | | **Summary** | |
| *IL13RA1* | interleukin 13 receptor, alpha 1 | | The protein encoded by this gene is a subunit of the interleukin 13 receptor. This protein has been shown to bind tyrosine kinase TYK2, and thus may mediate the signaling processes that lead to the activation of JAK1, STAT3 and STAT6 induced by IL13 and IL4. | |
| *IL21R* | interleukin 21 receptor | | The protein encoded by this gene is a cytokine receptor for interleukin 21 (IL21). This receptor transduces the growth promoting signal of IL21, and is important for the proliferation and differentiation of T cells, B cells, and natural killer (NK) cells. The ligand binding of this receptor leads to the activation of multiple downstream signaling molecules, including JAK1, JAK3, STAT1, and STAT3. | |
| *IL1A* | interleukin 1, alpha | | The protein encoded by this gene is a member of the interleukin 1 cytokine family. This cytokine is a pleiotropic cytokine involved in various immune responses, inflammatory processes, and hematopoiesis. | |
| *IL6* | interleukin 6 (interferon, beta 2) | | This gene encodes a cytokine that functions in inflammation and the maturation of B cells. The protein is primarily produced at sites of acute and chronic inflammation, where it is secreted into the serum and induces a transcriptional inflammatory response through interleukin 6 receptor, alpha. The functioning of this gene is implicated in a wide variety of inflammation-associated disease states. | |
|  | | | | |
| **Up-regulated interleukin at 15 minutes p.i.** | | | | |
| **Gene** | | **Description** | | **Summary** |
| *IL23A* | | interleukin 23, alpha subunit p19 | | This gene encodes a subunit of the heterodimeric cytokine interleukin 23 (IL23). IL23 can activate the transcription activator STAT4, and stimulate the production of interferon-gamma (IFNG). |
| *CXCR2* | | chemokine (C-X-C motif) receptor 2 | | The protein encoded by this gene is a member of the G-protein-coupled receptor family. This protein is a receptor for interleukin 8 (IL8). This receptor mediates neutrophil migration to sites of inflammation. The angiogenic effects of IL8 in intestinal microvascular endothelial cells are found to be mediated by this receptor |
| *IL10RB* | | interleukin 10 receptor, beta | | The protein encoded by this gene belongs to the cytokine receptor family. It is an accessory chain essential for the active interleukin 10 receptor complex. Coexpression of this and IL10RA proteins has been shown to be required for IL10-induced signal transduction. |
| *IL2* | | interleukin 2 | | The protein encoded by this gene is a secreted cytokine that is important for the proliferation of T and B lymphocytes. |
| *IL1R2* | | interleukin 1 receptor, type II | | The protein encoded by this gene is a cytokine receptor that belongs to the interleukin 1 receptor family. This protein binds interleukin alpha (IL1A), interleukin beta (IL1B), and interleukin 1 receptor, type I(IL1R1/IL1RA), and acts as a decoy receptor that inhibits the activity of its ligands. |
| *IL5* | | interleukin 5 (colony-stimulating factor, eosinophil) | | The protein encoded by this gene is a cytokine that acts as a growth and differentiation factor for both B cells and eosinophils. This cytokine is a main regulator of eosinopoiesis, eosinophil maturation and activation. |
| *IL2RG* | | interleukin 2 receptor, gamma | | The protein encoded by this gene is an important signaling component of many interleukin receptors, including those of interleukin -2, -4, -7 and -21, and is thus referred to as the common gamma chain. |
| *IL15RA* | | interleukin 15 receptor, alpha | | This gene encodes a cytokine receptor that specifically binds interleukin 15 (IL15) with high affinity. The receptors of IL15 and IL2. This receptor is reported to enhance cell proliferation and expression of apoptosis inhibitor BCL2L1/BCL2-XL and BCL2. |
| *IL4R* | | interleukin 4 receptor | | This gene encodes the alpha chain of the interleukin-4 receptor, a type I transmembrane protein that can bind interleukin 4 and interleukin 13 to regulate IgE production. The encoded protein also can bind interleukin 4 to promote differentiation of Th2 cells. |
| *IL7* | | interleukin 7 | | The protein encoded by this gene is a cytokine important for B and T cell development. This cytokine and the hepatocyte growth factor (HGF) form a heterodimer that functions as a pre-pro-B cell growth-stimulating factor. This cytokine can be produced locally by intestinal epithelial and epithelial goblet cells, and may serve as a regulatory factor for intestinal mucosal lymphocytes. |
| *IL6R* | | interleukin 6 receptor | | This gene encodes a subunit of the interleukin 6 (IL6) receptor complex. Interleukin 6 is a potent pleiotropic cytokine that regulates cell growth and differentiation and plays an important role in the immune response. |
| *IL22RA1* | | interleukin 22 receptor, alpha 1 | | The protein encoded by this gene belongs to the class II cytokine receptor family, and has been shown to be a receptor for interleukin 22 (IL22). |
| *IL28RA* | | interleukin 28 receptor, alpha (interferon, lambda receptor) | | The protein encoded by this gene belongs to the class II cytokine receptor family. This protein forms a receptor complex with interleukine 10 receptor, beta (IL10RB). The receptor complex has been shown to interact with three closely related cytokines, including interleukin 28A (IL28A), interleukin 28B (IL28B), and interleukin 29 (IL29). The expression of all three cytokines can be induced by viral infection |
| *IL15* | | interleukin 15 | | The protein encoded by this gene is a cytokine that regulates T and natural killer cell activation and proliferation. This cytokine and interleukine 2 share many biological activities. This cytokine induces the activation of JAK kinases, as well as the phosphorylation and activation of transcription activators STAT3, STAT5, and STAT6. |
| *IL3* | | interleukin 3 | | The protein encoded by this gene is a potent growth promoting cytokine. This cytokine is capable of supporting the proliferation of a broad range of hematopoietic cell types. |
| *IL4* | | interleukin 4 | | The protein encoded by this gene is a pleiotropic cytokine produced by activated T cells. This cytokine is a ligand for interleukin 4 receptor. The interleukin 4 receptor also binds to IL13, which may contribute to many overlapping functions of this cytokine and IL13. STAT6, a signal transducer and activator of transcription, has been shown to play a central role in mediating the immune regulatory signal of this cytokine. |
| *IL12A* | | interleukin 12A (natural killer cell stimulatory factor 1, cytotoxic lymphocyte maturation factor 1, p35) | | This gene encodes a subunit of a cytokine that acts on T and natural killer cells, and has a broad array of biological activities. The cytokine is a disulfide-linked heterodimer composed of the 35-kD subunit encoded by this gene, and a 40-kD subunit that is a member of the cytokine receptor family. This cytokine is required for the T-cell-independent induction of interferon (IFN)-gamma, and is important for the differentiation of both Th1 and Th2 cells. The responses of lymphocytes to this cytokine are mediated by the activator of transcription protein STAT4. |
| *IL1RAP* | | interleukin 1 receptor accessory protein | | Interleukin 1 induces synthesis of acute phase and proinflammatory proteins during infection, tissue damage, or stress, by forming a complex at the cell membrane with an interleukin 1 receptor and an accessory protein. This gene encodes the interleukin 1 receptor accessory protein. The protein is a necessary part of the interleukin 1 receptor complex which initiates signalling events that result in the activation of interleukin 1-responsive genes. |
