# Supplementary material for: Systems Biology Analysis of Brucella Infected Peyer's Patch Reveals Rapid Invasion with Modest Transient Perturbations of the Host Transcriptome
Source: PLoS One. 2013 Dec 9;8(12):e81719. doi: 10.1371/journal.pone.0081719 (PMC3857238; doi:10.1371/journal.pone.0081719)
Supplement: File S1 — Supplemental figures and tables. Figure A in File S1. Validation of Bovine Microarray Results by Quantitative Real Time-PCR. cDNA was synthesized from the same RNA samples used for microarray hybridization. Five randomly selected genes (A = BPI; B = MAPK1; C = MIF; D = CCL2; E = IL8.) that were differentially expressed by microarrays in B. melitensis-infected bovine Peyer's patch between 15 min and 4 h p.i. as compared to non-infected tissues (control) extracted at the same time points, were validated by quantitative RT-PCR. Fold changes was normalized to the expression of GAPDH and calculated using the ΔΔCt method. All tested genes at all time points had fold-changes altered in the same direction in microarray and qRT-PCR. White bars represent fold-change by microarray analysis and black bars represent fold-change by qRT-PCR. Table S1 in File S1. Detailed List of Host Genes with Differential Expression (z-score >|2.24|) in B. melitensis Infected vs. Control Bovine Jejunal-Ileal Peyer's Patch in at least one time point. Black numbers in the body of the table indicate differentially expressed (activated: (+) numbers; repressed: (−) numbers) while red numbers represent non-differentially expressed genes. Table S2 in File S1. Bayesian z-score for All Host Pathways in B. melitensis Infected vs. Control Bovine Jejunal-Ileal Peyer's Patch. Black numbers in the body of the table indicate differentially expressed (activated: (+) numbers; repressed: (−) numbers) while red numbers represent non-differentially expressed genes. Table S3 in File S1. List of All Biological Process-Related Host Genes Differentially Expressed in B. melitensis Infected vs. Control Bovine Jejunal-Ileal Peyer's Patch. Black numbers in the body of the table indicate differentially expressed (activated: (+) numbers; repressed: (−) numbers) while red numbers represent non-differentially expressed genes. Table S4 in File S1. List of All Cellular Component-Related Host Genes Differentially Expressed in B. [file pone.0081719.s001.zip › MS Bmel Final Suppl File Figure Tables 31x2013/Table S24_Tight_Junction mech genes.doc]

| *NRAS* | neuroblastoma RAS viral (v-ras) oncogene homolog | Gene that encodes a membrane protein that shuttles between the Golgi apparatus and the plasma membrane. This shuttling is regulated through palmitoylation and depalmitoylation by the ZDHHC9-GOLGA7 complex. The encoded protein, which has intrinsic GTPase activity, is activated by a guanine nucleotide-exchange factor and inactivated by a GTPase activating protein. |
| --- | --- | --- |
| *SPTAN1* | spectrin, alpha, non-erythrocytic 1 | Spectrins are a family of filamentous cytoskeletal proteins that function as essential scaffold proteins that stabilize the plasma membrane and organize intracellular organelles. This gene encodes an alpha spectrin that is specifically expressed in nonerythrocytic cells. The encoded protein has been implicated in other cellular functions including DNA repair and cell cycle regulation. |
| *PRKCG* | protein kinase C, gamma | Protein kinase C (PKC) is a family of serine- and threonine-specific protein kinases that can be activated by calcium and second messenger diacylglycerol. PKC family members phosphorylate a wide variety of protein targets and are known to be involved in diverse cellular signaling pathways. |
| *PPP2R2A* | protein phosphatase 2, regulatory subunit B, alpha | This gene encodes a protein that belongs to the phosphatase 2 regulatory subunit B family. Protein phosphatase 2 is one of the four major Ser/Thr phosphatases, and it is implicated in the negative control of cell growth and division. It consists of a common heteromeric core enzyme, which is composed of a catalytic subunit and a constant regulatory subunit, that associates with a variety of regulatory subunits |
| *EPB41* | erythrocyte membrane protein band 4.1 | The protein encoded by this gene, together with spectrin and actin, constitute the red cell membrane cytoskeletal network. This complex plays a critical role in erythrocyte shape and deformability. |
| *PTEN* | phosphatase and tensin homolog | The protein encoded this gene is a phosphatidylinositol-3,4,5-trisphosphate 3-phosphatase. It contains a tensin like domain as well as a catalytic domain similar to that of the dual specificity protein tyrosine phosphatases. Unlike most of the protein tyrosine phosphatases, this protein preferentially dephosphorylates phosphoinositide substrates. It negatively regulates intracellular levels of phosphatidylinositol-3,4,5-trisphosphate in cells and functions as a tumor suppressor by negatively regulating AKT/PKB signaling pathway. |
| *CSNK2B* | casein kinase 2, beta polypeptide | This gene encodes the beta subunit of casein kinase II, a ubiquitous protein kinase which regulates metabolic pathways, signal transduction, transcription, translation, and replication. |
| *YES1* | v-yes-1 Yamaguchi sarcoma viral oncogene homolog 1 | The encoded protein has tyrosine kinase activity and belongs to the src family of proteins. |
| *RHOA* | ras homolog family member A | Regulates a signal transduction pathway linking plasma membrane receptors to the assembly of focal adhesions and actin stress fibers. Involved in a microtubule-dependent signal that is required for the myosin contractile ring formation during cell cycle cytokinesis. Plays an essential role in cleavage furrow formation. Required for the apical junction formation of keratinocyte cell-cell adhesion. |
| *CSNK2A1* | casein kinase 2, alpha 1 polypeptide | Catalytic subunit of a constitutively active serine/threonine-protein kinase complex that phosphorylates a large number of substrates containing acidic residues C-terminal to the phosphorylated serine or threonine. Regulates numerous cellular processes, such as cell cycle progression, apoptosis and transcription, as well as viral infection. May act as a regulatory node which integrates and coordinates numerous signals leading to an appropriate cellular response. |
| *MYL5* | myosin, light chain 5, regulatory | Myosins are a large family of motor proteins that share the common features of ATP hydrolysis, actin binding and potential for kinetic energy transduction. |
| *CGN* | cingulin | Probably plays a role in the formation and regulation of the tight junction (TJ) paracellular permeability barrier |
| *CLDN1* | claudin 1 | Plays a major role in tight junction-specific obliteration of the intercellular space, through calcium-independent cell-adhesion activity |
| *CDC42* | cell division cycle 42 (GTP binding protein, 25kDa) | The protein encoded by this gene is a small GTPase of the Rho-subfamily, which regulates signaling pathways that control diverse cellular functions including cell morphology, migration, endocytosis and cell cycle progression. Involved in epithelial cell polarization processes. |
| *AKT3* | v-akt murine thymoma viral oncogene homolog 3 (protein kinase B, gamma) | AKT3 is one of 3 closely related serine/threonine-protein kinases (AKT1, AKT2 and AKT3) called the AKT kinase, and which regulate many processes including metabolism, proliferation, cell survival, growth and angiogenesis. |

**Table S24. Tight Junction Pathway - Dominant Down-Regulated Mechanistic Genes.**
