# Supplementary material for: Systems Biology Analysis of Brucella Infected Peyer's Patch Reveals Rapid Invasion with Modest Transient Perturbations of the Host Transcriptome
Source: PLoS One. 2013 Dec 9;8(12):e81719. doi: 10.1371/journal.pone.0081719 (PMC3857238; doi:10.1371/journal.pone.0081719)
Supplement: File S1 — Supplemental figures and tables. Figure A in File S1. Validation of Bovine Microarray Results by Quantitative Real Time-PCR. cDNA was synthesized from the same RNA samples used for microarray hybridization. Five randomly selected genes (A = BPI; B = MAPK1; C = MIF; D = CCL2; E = IL8.) that were differentially expressed by microarrays in B. melitensis-infected bovine Peyer's patch between 15 min and 4 h p.i. as compared to non-infected tissues (control) extracted at the same time points, were validated by quantitative RT-PCR. Fold changes was normalized to the expression of GAPDH and calculated using the ΔΔCt method. All tested genes at all time points had fold-changes altered in the same direction in microarray and qRT-PCR. White bars represent fold-change by microarray analysis and black bars represent fold-change by qRT-PCR. Table S1 in File S1. Detailed List of Host Genes with Differential Expression (z-score >|2.24|) in B. melitensis Infected vs. Control Bovine Jejunal-Ileal Peyer's Patch in at least one time point. Black numbers in the body of the table indicate differentially expressed (activated: (+) numbers; repressed: (−) numbers) while red numbers represent non-differentially expressed genes. Table S2 in File S1. Bayesian z-score for All Host Pathways in B. melitensis Infected vs. Control Bovine Jejunal-Ileal Peyer's Patch. Black numbers in the body of the table indicate differentially expressed (activated: (+) numbers; repressed: (−) numbers) while red numbers represent non-differentially expressed genes. Table S3 in File S1. List of All Biological Process-Related Host Genes Differentially Expressed in B. melitensis Infected vs. Control Bovine Jejunal-Ileal Peyer's Patch. Black numbers in the body of the table indicate differentially expressed (activated: (+) numbers; repressed: (−) numbers) while red numbers represent non-differentially expressed genes. Table S4 in File S1. List of All Cellular Component-Related Host Genes Differentially Expressed in B. [file pone.0081719.s001.zip › MS Bmel Final Suppl File Figure Tables 31x2013/Table S25_Trefoil mech genes.doc]

| *PTK2* | PTK2 protein tyrosine kinase 2 | Non-receptor protein-tyrosine kinase that plays an essential role in regulating cell migration, adhesion, spreading, reorganization of the actin cytoskeleton, formation and disassembly of focal adhesions and cell protrusions, cell cycle progression, cell proliferation and apoptosis. |
| --- | --- | --- |
| *GHR* | growth hormone receptor | This gene encodes a member of the type I cytokine receptor family, which is a transmembrane receptor for growth hormone. Binding of growth hormone to the receptor leads to receptor dimerization and the activation of an intra- and intercellular signal transduction pathway leading to growth. |
| *CTNNB1* | catenin (cadherin-associated protein), beta 1, 88kDa | The protein encoded by this gene is part of a complex of proteins that constitute adherens junctions (AJs). AJs are necessary for the creation and maintenance of epithelial cell layers by regulating cell growth and adhesion between cells. The encoded protein also anchors the actin cytoskeleton and may be responsible for transmitting the contact inhibition signal that causes cells to stop dividing once the epithelial sheet is complete. |
| *MAPK3* | mitogen-activated protein kinase 3 | Serine/threonine kinase which acts as an essential component of the MAP kinase signal transduction pathway.MAPK1/ERK2 and MAPK3/ERK1 are the 2 MAPKs which play an important role in the MAPK/ERK cascade. |
| *SHC1* | SHC (Src homology 2 domain containing) transforming protein 1 | Signaling adapter that couples activated growth factor receptors to signaling pathways. Participates in signaling downstream of the angiopoietin receptor TEK/TIE2, and plays a role in the regulation of endothelial cell migration and sprouting angiogenesis |

**Table S25. Trefoil Factors Initiated Mucosal Healing - Dominant Down-Regulated Mechanistic Genes.**
